# METTL3-mediated *N*^*6*^-methyladenosine mRNA modification enhances long-term memory consolidation

**DOI:** 10.1038/s41422-018-0092-9

**Published:** 2018-10-08

**Authors:** Zeyu Zhang, Meng Wang, Dongfang Xie, Zenghui Huang, Lisha Zhang, Ying Yang, Dongxue Ma, Wenguang Li, Qi Zhou, Yun-Gui Yang, Xiu-Jie Wang

**Affiliations:** 1 Key Laboratory of Genetic Network Biology, Institute of Genetics and Developmental Biology, Chinese Academy of Sciences, 100101 Beijing, China; 2 University of Chinese Academy of Sciences, 100049 Beijing, China; 3 Key Laboratory of Genomic and Precision Medicine, Collaborative Innovation Center of Genetics and Development, Beijing Institute of Genomics, Chinese Academy of Sciences, 100101 Beijing, China; 4 State Key Laboratory of Stem Cell and Reproductive Biology, Institute of Zoology, Chinese Academy of Sciences, 100101 Beijing, China; 5 Institute for Stem Cell and Regeneration, Chinese Academy of Sciences, 100101 Beijing, China

## Abstract

The formation of long-term memory is critical for learning ability and social behaviors of humans and animals, yet its underlying mechanisms are largely unknown. We found that the efficacy of hippocampus-dependent memory consolidation is regulated by METTL3, an RNA *N*^6^-methyladenosine (m^6^A) methyltransferase, through promoting the translation of neuronal early-response genes. Such effect is exquisitely dependent on the m^6^A methyltransferase function of METTL3. Depleting METTL3 in mouse hippocampus reduces memory consolidation ability, yet unimpaired learning outcomes can be achieved if adequate training was given or the m^6^A methyltransferase function of METTL3 was restored. The abundance of METTL3 in wild-type mouse hippocampus is positively correlated with learning efficacy, and overexpression of METTL3 significantly enhances long-term memory consolidation. These findings uncover a direct role of RNA m^6^A modification in regulating long-term memory formation, and also indicate that memory efficacy difference among individuals could be compensated by repeated learning.

## Introduction

Long-term memory, as accumulated with rote learning or experience, is essential for mammalian behavioral adaptation and intelligence development. The formation of long-term memory involves several brain regions, including hippocampus, prefrontal cortex, and amygdala. Hippocampus is a small yet major brain region locates under cerebral cortex, and plays a major role in the consolidation of short-term memory to long-term memory.

It has been proven that the transformation from short-term memories to long-term memories requires *de novo* protein synthesis for synaptic consolidation, of which long-term potentiation (LTP) of neurons is considered as one of the major contributors.^1–3^ The formation of long-term memory is coupled with rapid and transient expression of immediate early genes (IEGs), including *Arc*, *c-Fos*, *Egr1*, *Npas4* and *Nr4a1*. Studies have proven IEGs as the essential mediators for long-term memory; deficiency in IEG expression results in impaired learning ability and memory formation.^4^ Most IEGs are DNA binding proteins (e.g. *c-Fos*, *Egr1* and *Npas4*) that can activate downstream neurotrophic factors to modulate synaptic plasticity,^5^ yet how such process is regulated remains largely elusive. *N*^6^-methyladenosine (m^6^A), the most abundant modification on mRNAs in eukaryotic cells, plays important roles in various biological progresses through regulating mRNA metabolism.^6^ The presence of m^6^A on mRNAs is reversible, catalyzed by a protein complex with METTL3 as the key methyltransferase and removed by demethylases FTO and ALKBH5.^6^ Several previous studies have implied that m^6^A may relate to the memory process,^7,8^ yet the detailed functions and mechanisms of m^6^A in regulating memory formation remain to be characterized.

Here, using *CaMKIIα-Cre* mediated *Mettl3* conditional knockout mice, we have found that METTL3-catalyzed m^6^A formation enhances long-term memory consolidation, likely through translational regulation of learning related genes. Unexpectedly, although mice with higher METTL3 level learn faster, the function of m^6^A can be compensated by additional training, demonstrating a new layer of robustness of memory formation.

## Results

### Postnatal deletion of *Mettl3* in hippocampus caused no morphological defects but prolonged the process of long-term memory consolidation

**Fig. 1:**
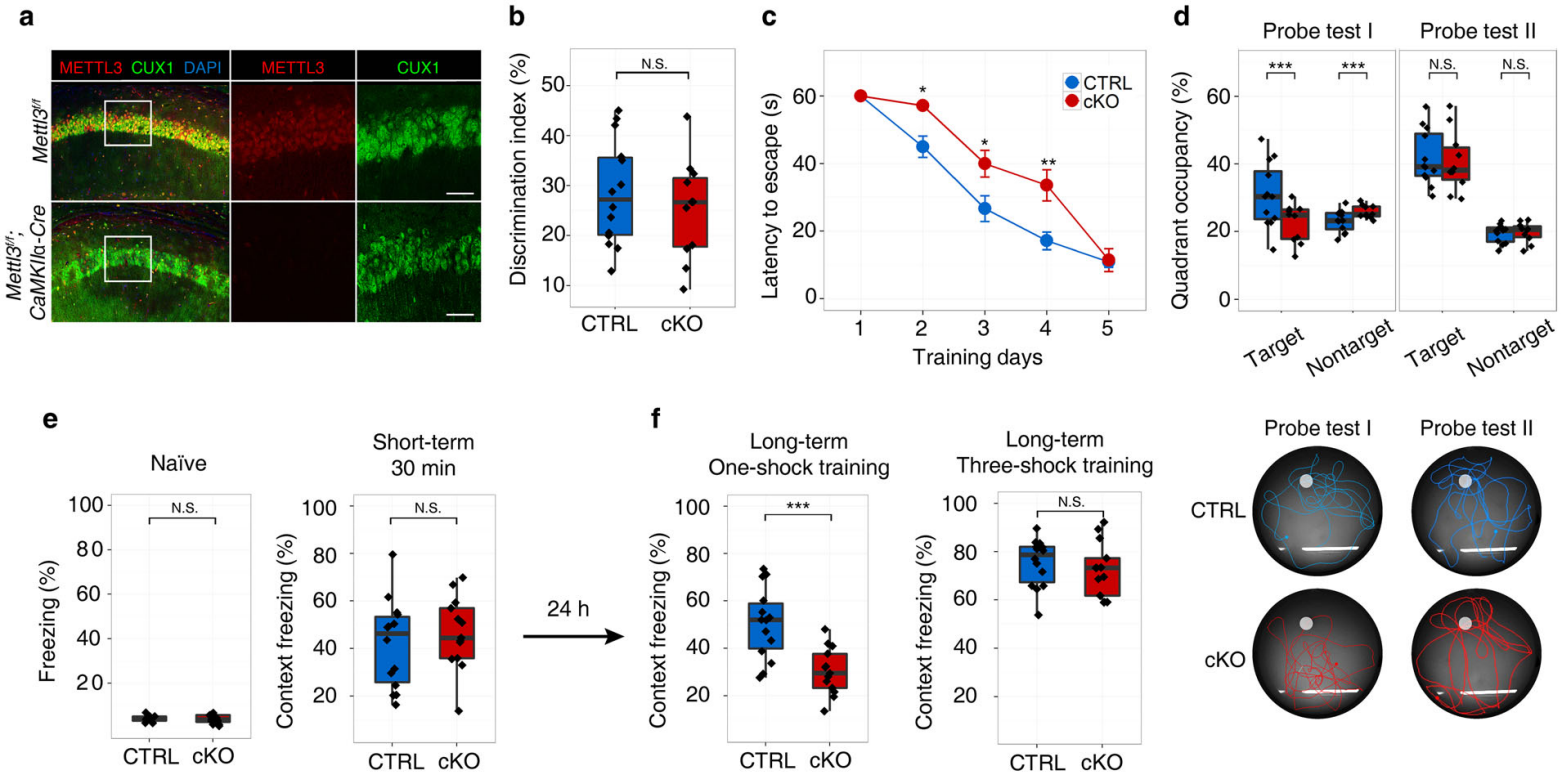
Postnatal deletion of *Mettl3* in hippocampus prolonged the process of long-term memory consolidation. **a** Characterization of *Mettl3* conditional knockout in the CA1 region of 8-week male mouse brains. Scale bars, 100 µm. **b** Discrimination index of the control (CTRL, *Mettl3*^*f/f*^) and *Mettl3* conditional knockout (cKO, *Mettl3*^*f/f*^*; CaMKIIα-Cre*) mice in novel object recognition test (CTRL, *n* = 13 mice; cKO, *n* = 10 mice). **c** Results of Morris water maze test on five consecutive training days. Group difference was measured by two-way repeated ANOVA, *P* < 0.001. **d** Probe tests of the CTRL (blue) and cKO (red) mice during the Morris water maze test. Upper panel, occupancy frequency for the target and nontarget quadrants; lower panel, representative swimming paths of the upper panel. **e** Freezing behavior before (naïve) and 30 min (short-term) after one-shock fear conditioning. **f** Freezing behavior during contextual test 24 h after one-shock training (left) or three-shock training (right). In (**d**), (**e**), and (**f**), CTRL, *n* = 14 mice; cKO, *n* = 13 mice. Student’s *t*-test, *P < 0.05, **P < 0.01, ***P < 0.001, N.S., not significant

To explore the function of m^6^A in regulating memory formation, we first crossed *Mettl3*^*flox/flox*^ mice with *CaMKIIα-Cre* mice to generate forebrain excitatory neuron-specific *Mettl3* conditional knockout mice (*Mettl3*^*flox/flox*^*; CaMKIIα-Cre*, hereafter cKO) (Fig. 1a and Supplementary Fig. S1a). The cKO mice were viable, fertile and developed normally into adulthood with body and brain weights comparable to the control littermates (*Mettl3*^*flox/flox*^, hereafter CTRL) (Supplementary Fig. S1b and c). Histological analysis revealed that 8-week-old cKO mice had normal brain architecture and no observable apoptosis (Supplementary Fig. S1d to h). Rotarod test, open field test, elevated plus maze test and Morris water maze test all detected no difference between the CTRL and cKO mice in terms of motor coordination, exploratory behavior, anxiety levels, and swimming ability (Supplementary Fig. S2a to d). Moreover, cKO mice showed intact short-term memory in new object recognition test (Fig. 1b and Supplementary Fig. S2e). We next examined the hippocampus-dependent long-term memory formation in cKO mice by Morris water maze test.^9^ Interestingly, although the cKO mice took more time than the CTRL group to find the hidden platform in the initial training days, they can still learn gradually. On day 5, the cKO mice reached the platform as fast as the CTRL ones (Fig. 1c). Consistently, the cKO mice spent significantly less amount of time within the target quadrant than the CTRL ones during the first probe test after training day 3, but showed no difference from the CTRL mice in the second probe test after training day 5 (Fig. 1d). In the fear conditioning test, the cKO mice behaved as good as the CTRL ones in contextual freezing evaluation before and 30 min after one mild foot electric shock (Fig. 1e), suggesting that the cKO mice had normal peripheral pain perception and short-term memory. However, in the contextual test conducted 24 h after the first shock, the cKO mice froze only half of the duration as the CTRL mice did, indicating a deficiency in long-term memory formation (Fig. 1f). But prolonged training with three mild foot electric shocks increased the duration of contextual freezing behavior of both the CTRL and cKO mice to a similar level (Fig. 1f), which is in concert with the water maze test. Collectively, these data suggest that depletion of METTL3 in hippocampus resulted in decreased long-term memory formation ability in mice, but did not alter the final learning outcome when adequate training was provided.

### Postnatal deletion of *Mettl3* in hippocampus impairs long-term potentiation without affecting neuronal intrinsic electric properties and short-term plasticity

**Fig. 2:**
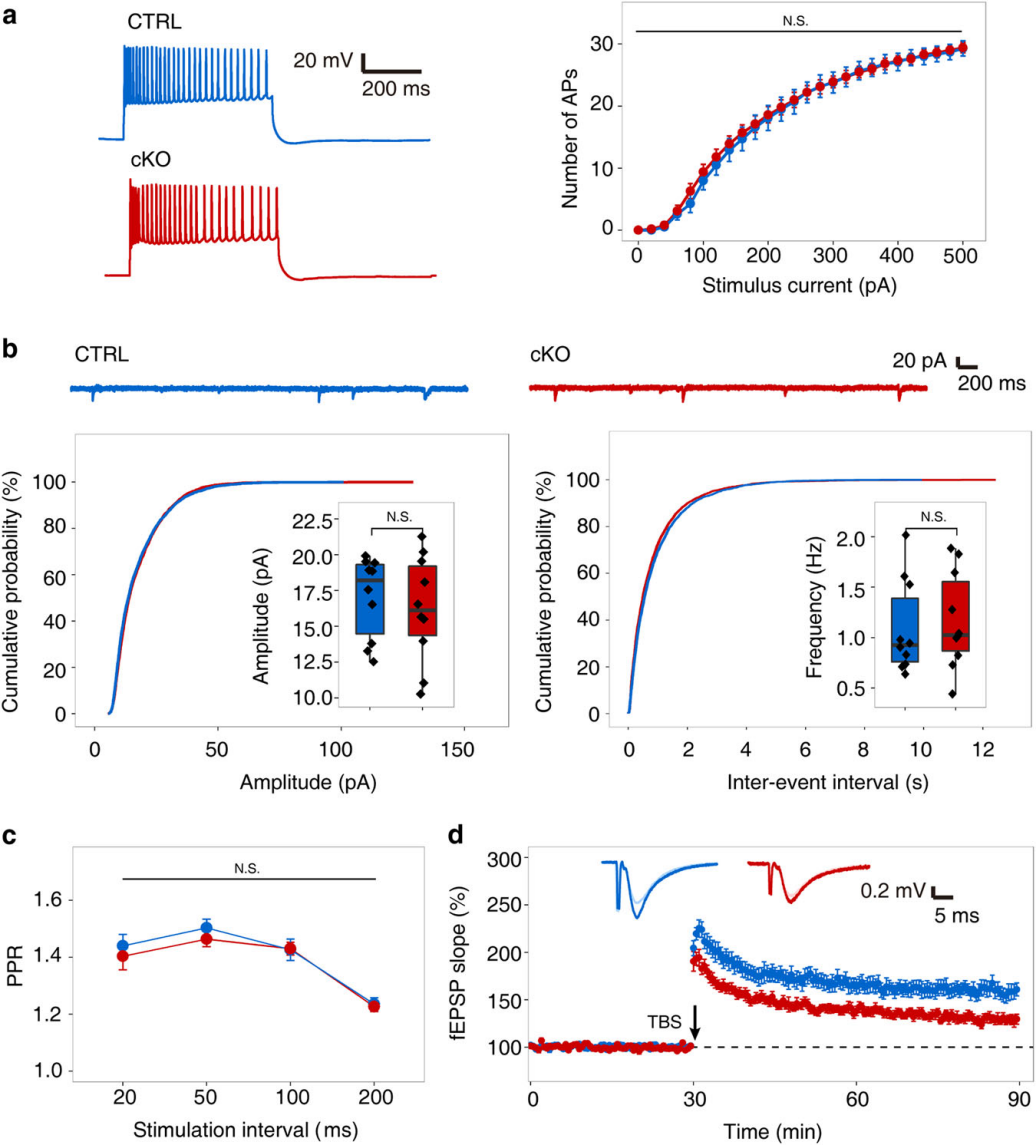
Electrophysiological tests of *Mettl3*-depleted hippocampus. **a** Representative traces (left) and numbers of action potentials (right) responding to stimuli with different intensity in CTRL (blue) and cKO (red) CA1 pyramidal neurons (*n* = 10 hippocampal slices from 3 mice per group). **b** Representative traces of mESPCs (top) and distribution of cumulative probability of mEPSCs amplitude (bottom left) and frequency (bottom right) of the CTRL (blue) and cKO (red) CA1 pyramidal neurons. The insets show the comparison of mean values. **c** Paired-pulse ratio at different inter-stimulus intervals in CTRL (blue) and cKO (red) groups (*n* = 9 hippocampal slices from 3 mice per group). **d** Field EPSP slope change in CTRL (blue) and cKO (red) groups following a single theta-burst stimulation (TBS). Group difference was measured by two-way repeated ANOVA, *P* < 0.001. Insets show representative traces at baseline (light blue and red lines for CTRL and cKO, respectively) and 1 h (blue and red lines for CTRL and cKO, respectively) after TBS induction (*n* = 9 hippocampal slices from 3 mice per group). Student’s *t*-test, N.S., not significant

To investigate the physiological cause of the above phenotype, we performed whole-cell patch clamp on CA1 pyramidal neurons of both the CTRL and cKO mice. The CA1 pyramidal neurons of the cKO mice are all normal in terms of resting membrane potential, membrane resistance, firing rate, amplitude and duration, as well as response to injected currents (Fig. 2a and Supplementary Fig. S3a). We next tested the excitatory synaptic transmission ability of CA1 pyramidal neurons by measuring the miniature excitatory post-synaptic currents (mEPSCs) and synaptic strength by calculating the input-output relationship (I/O), and observed no significant change in cKO neurons (Fig. 2b, and Supplementary Fig. S3b). Paired-pulse facilitation was also unaltered in cKO pyramidal neurons, suggesting the cells had normal short-term synaptic plasticity (Fig. 2c). However, long-term potentiation (LTP) on hippocampal Schaffer collateral pathway exhibited a significant decrease in the slope of field excitatory postsynaptic potential (fEPSP) (Fig. 2d and Supplementary Fig. S3c), which was reported to be sufficient to cause long-term memory deficiency.^10^ Consistent with the electrophysiological results, the abundance of key synaptic proteins (GluR1, GluR2, GluN, GluN2A, GluN2B, CAMKIIA, PSD95, HOMER1, and SHANK1) isolated by synaptosomal fractionation all showed no difference between the CTRL and cKO mice in hippocampus (Supplementary Fig. S4), supporting the normal short-term synaptic plasticity of cKO pyramidal neurons.

### METTL3-mediated long-term memory formation is dependent on its m^6^A methyltransferase function

**Fig. 3:**
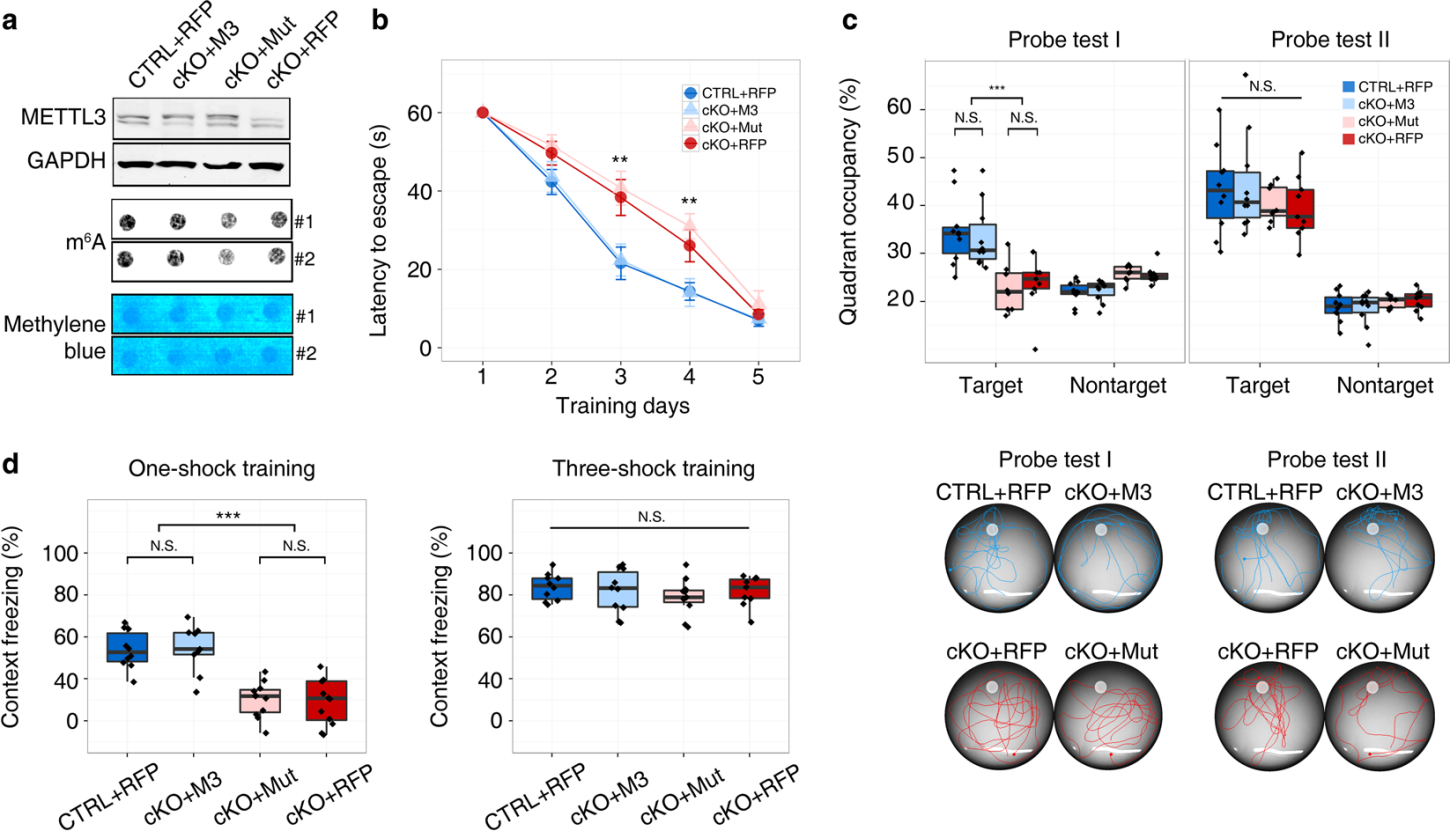
METTL3 regulates long-term memory formation via its m^6^A methyltransferase function. **a** Restoration of METTL3 and m^6^A in the hippocampus of cKO mice. #1 and #2 represent two biological replicates. **b** Re-introducing wildtype *Mettl3* (M3), but not *Mettl3* with deficient m^6^A methyltransferase function (Mut), to hippocampus rescued the learning delay of cKO mice in Morris water maze test. Group difference was measured by two-way repeated ANOVA, *P* < 0.001. **c** Probe tests of groups in (**b**). Upper panel, occupancy frequency in the target and nontarget quadrants; lower panel, representative swimming paths of the upper panel. In (**b**) and (**c**), RFP: injection control; CTRL + RFP, *n* *=* 10 mice; cKO + M3, *n* = 10 mice; cKO + Mut, *n* = 8 mice; cKO + RFP, *n* = 9 mice. **d** Re-introducing wildtype *Mettl3* rescues the learning defect of cKO mice in fear-conditioning test (CTRL + RFP, *n* = 10 mice; cKO + M3, *n* = 9 mice; cKO + Mut, *n* = 10 mice; cKO + RFP, *n* = 10 mice). In (**c**) and (**d**), ANOVA and Tukey’s HSD *post hoc* test, *P < 0.05, **P < 0.01, ***P < 0.001, N.S., not significant

To characterize whether the METTL3 depletion-associated long-term memory deficiency is indeed caused by the m^6^A modification function of METTL3, we performed a rescue experiment by stereotaxically injecting serotype 2/DJ adenosine-associated virus (AAV2/DJ) carrying mouse *Mettl3* cDNA sequence (M3) or *Mettl3* cDNA sequence with the methyltransferase domain-mutated^11,12^ (DPPW motif mutated to APPA, Mut) into the dorsal hippocampus of 7-week-old cKO mice (Supplementary Fig. S5). The same age CTRL and cKO mice injected with AAV2/DJ carrying red fluorescent protein (RFP) were used as the positive and negative controls, respectively. After 2 weeks of recovery, these four groups of mice were examined using Morris water maze test and fear conditioning test. As expected, both the cKO + M3 and cKO + Mut mice synthesized more METTL3 proteins in hippocampus compared to the cKO + RFP group, but increased m^6^A abundance was only detected in the cKO + M3 mice (Fig. 3a). Consequently, the cKO + M3 mice performed as good as the CTRL + RFP group in both the water maze and fear conditioning tests, but the cKO + Mut mice showed no improvement as compared with the cKO + RFP group (Fig. 3b–d), indicating that METTL3-related long-term memory formation depends on the m^6^A methyltransferase function of METTL3. Again, after 5 days of water maze training and 3 shocks in the fear conditioning training, all four groups of mice reached the same performance level (Fig. 3b–d), demonstrating the ability of cKO + Mut mice to form long-term memory after adequate training.

### m^6^A modification is dynamically regulated during memory consolidation

**Fig. 4:**
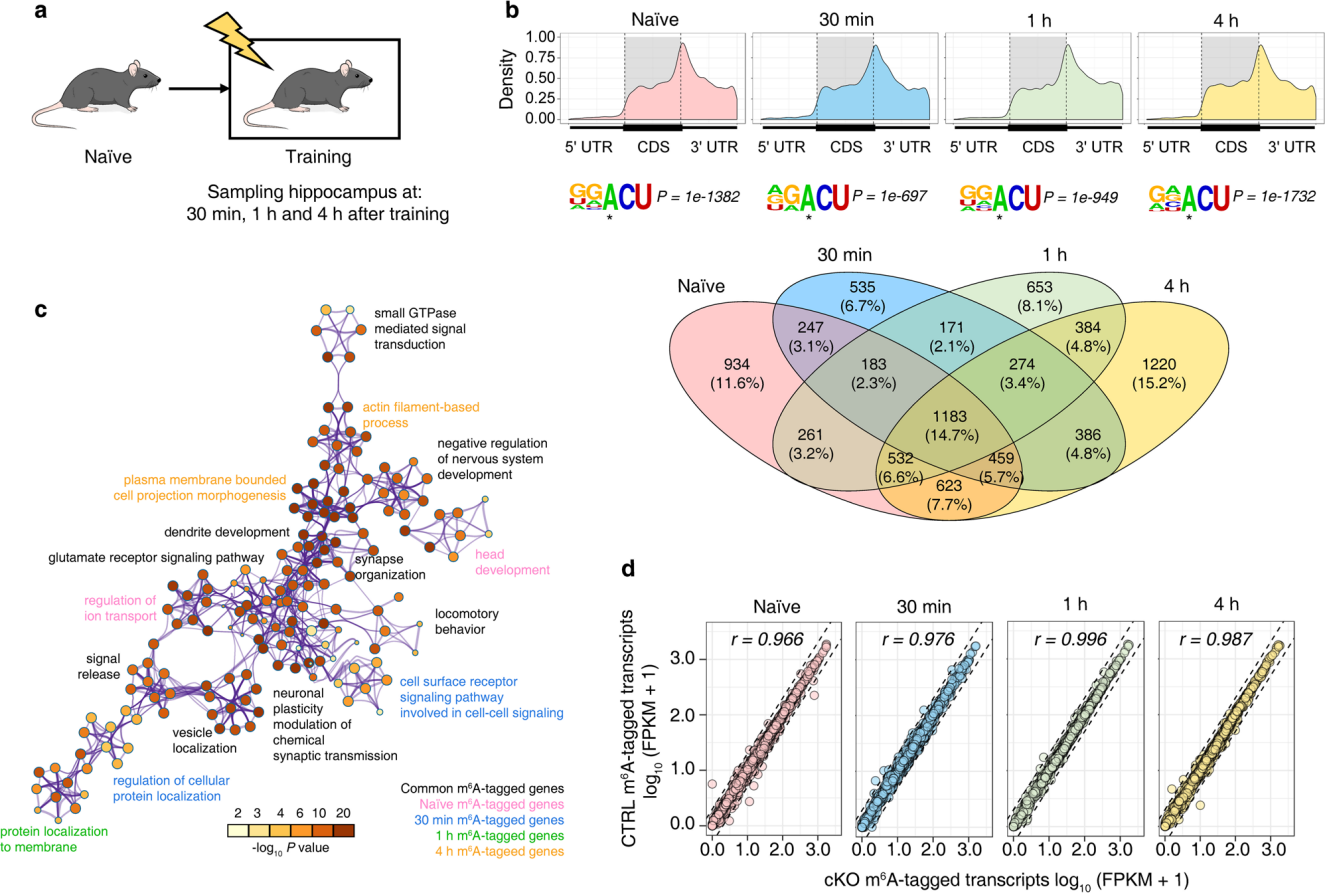
m^6^A methylome is dynamically regulated during memory consolidation. **a** Experimental design of sampling strategy. **b** m^6^A distribution (top panel), motif (middle panel) and number of m^6^A-tagged genes (down panel) before and after fear conditioning training. **c** Gene ontology (GO) enrichment analysis of common and timepoint specific m^6^A-tagged genes. Node size is proportionate to related gene numbers, color scales represent term enrichment significance. **d** Expression comparison of m^6^A-tagged genes between CTRL and cKO hippocampus at different timepoints. *r*, Pearson correlation coefficient

To unravel the molecular mechanisms underlying m^6^A-mediated memory consolidation, we performed single-base m^6^A methylome detection by miCLIP-m^6^A-seq^13,14^ using CTRL mice hippocampus tissues collected at 0 min (naïve), 30 min, 1 h, and 4 h after one-shock fear conditioning training (Fig. 4a), and identified a total of 8941, 5995, 6367 and 10853 m^6^A sites (with an RRACU motif and preferentially distributed around stop codons) corresponding to 4422, 3438, 3641, and 5061 expressed genes (referred as m^6^A-tagged genes below) at the above time points, respectively (Fig. 4b). Among them, 1183 genes were consistently modified at all time points. Gene ontology analysis revealed an enrichment of functions in synaptic signaling and neural development among m^6^A consistently modified genes, an enrichment of functions in membrane-related protein deposition among genes specifically modified by m^6^A at 30 min and 1 h post training, and an enrichment of functions in axon projection among genes specifically modified by m^6^A at 4 h post training (Fig. 4c), which were in concert with the memory consolidation mechanisms.^15,16^ It is worth to note that RNA-seq detected no significant expression difference of the m^6^A-tagged genes between CTRL and cKO mice hippocampus tissues collected at 0 min (naïve), 30 min, 1 h, and 4 h after one-shock fear conditioning training (Fig. 4d and Supplementary Fig. S6), indicating that the transcriptional regulation of cells in the cKO mouse hippocampi remains intact.

### Depletion of m^6^A resulted in insufficient translation of immediate-early genes

**Fig. 5:**
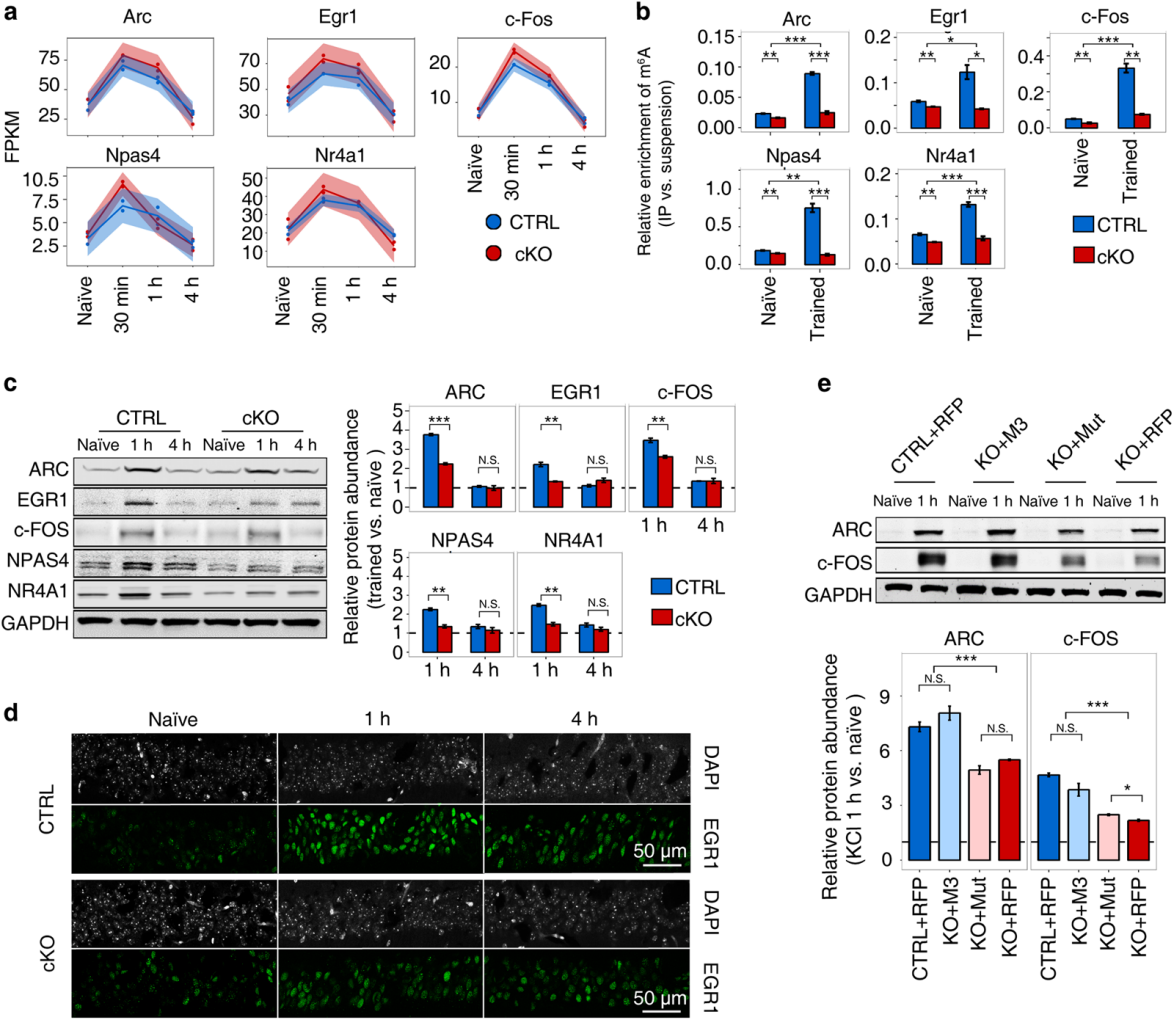
m^6^A promotes the translation of immediate-early genes upon activity induction. **a** Immediate-early genes (IEGs) are comparably induced by fear conditioning training in the hippocampus of both the CTRL (blue) and cKO (red) mice. **b** Increment of m^6^A modification abundance on IEG mRNAs after fear conditioning training in hippocampus samples of the CTRL mice but not the *Mettl3* cKO mice. IP, immunoprecipitated fraction. **c** Translation of IEGs is impaired in the cKO mice after training. **d** Immunofluorescent image of EGR1 in CA1 region before and after fear conditioning training (1 h and 4 h). **e** Translation of IEGs is impaired in *Mettl3*-KO primary cortical neurons, which can be rescued by re-expression of wildtype *Mettl3* but not mutated *Mettl3*. Student’s *t*-test, *P < 0.05, **P *<* 0.01, ***P *<* 0.001, N.S., not significant, (**a**) *n* = 2 replicates, (**b**), (**c**) and (**e**) *n* = 3 replicates

To explain the seemingly paradox between the functional defects and mRNA expression consistency among the CTRL and cKO mice, we examined the protein abundance of 5 well-studied IEGs (*Arc*, *Egr1*, *c-Fos*, *Npas4*, and *Nr4a1*), which should be rapidly activated after learning^17^ and are the upstream regulators for synaptic LTP and long-term memory formation,^4,18–23^ using western blot. The expression of these IEGs were rapidly induced in the CTRL hippocampus samples by fear conditioning training, with around 2-fold increment at the RNA level at 30 min post training (Fig. 5a). Significantly increased m^6^A modification abundance was detected on these genes in the CTRL samples after training (Fig. 5b and Supplementary Fig. S7a). Single-base resolution miCLIP-m^6^A-seq also identified new m^6^A modification sites on the transcripts of some IEGs after training, especially on the mRNAs of *Egr1* and *Npas4* (Supplementary Table S1), further confirming the dynamic m^6^A modification changes on these genes. However, although the expression of these IEGs in the cKO hippocampus was induced to the similar level as that in the CTRL ones at 30 min post training (Fig. 5a), transcripts of these IEGs lacked m^6^A modification in the cKO samples due to the absence of METTL3 (Fig. 5b). In concert with the known function of m^6^A modification,^24^ all these IEGs produced less proteins in cKO mice than in the CTRL ones (Fig. 5c, d), suggesting that the impaired LTP and prolonged learning process in cKO mice may due to insufficient protein synthesis in the absence of m^6^A. To further confirm this, we induced the expression of *Arc* and *c-Fos* in cultured primary cortical neurons via KCl treatment.^19^ Consistent with the in vivo data, the *Mettl3*-KO neurons produced less ARC and c-FOS proteins than the CTRL ones after KCl induction, but such difference was rescued by introducing wildtype METTL3 into KO neurons (Fig. 5e). Again, METTL3 with mutated methyltransferase domain failed to rescue the protein translation deficiency (Fig. 5e), indicating the essential roles of m^6^A methyltransferase function of METTL3 in such regulation. Overexpression (OE) of METTL3, but not METTL3 with mutated methyltransferase domain, in KCl treated primary cortical neurons also significantly enhanced the translation of *Arc* and *c-Fos*, as compared with the control groups (Supplementary Fig. S7b). On the other hand, no protein abundance change was detected for the translation initiation factor eIF2*α* and translation related m^6^A-binding protein YTHDF1^24^ under various METTL3 manipulation conditions in the hippocampus of *Mettl3* cKO or OE mice (Supplementary Fig. S7c). Together with the above mentioned normal synaptic protein abundance in *Mettl3* cKO mice (Supplementary Fig. S4), these results demonstrate that the altered IEG protein abundance in these samples is m^6^A dependent.

### METTL3 abundance correlates with learning efficacy and can enhance long-term memory formation

**Fig. 6:**
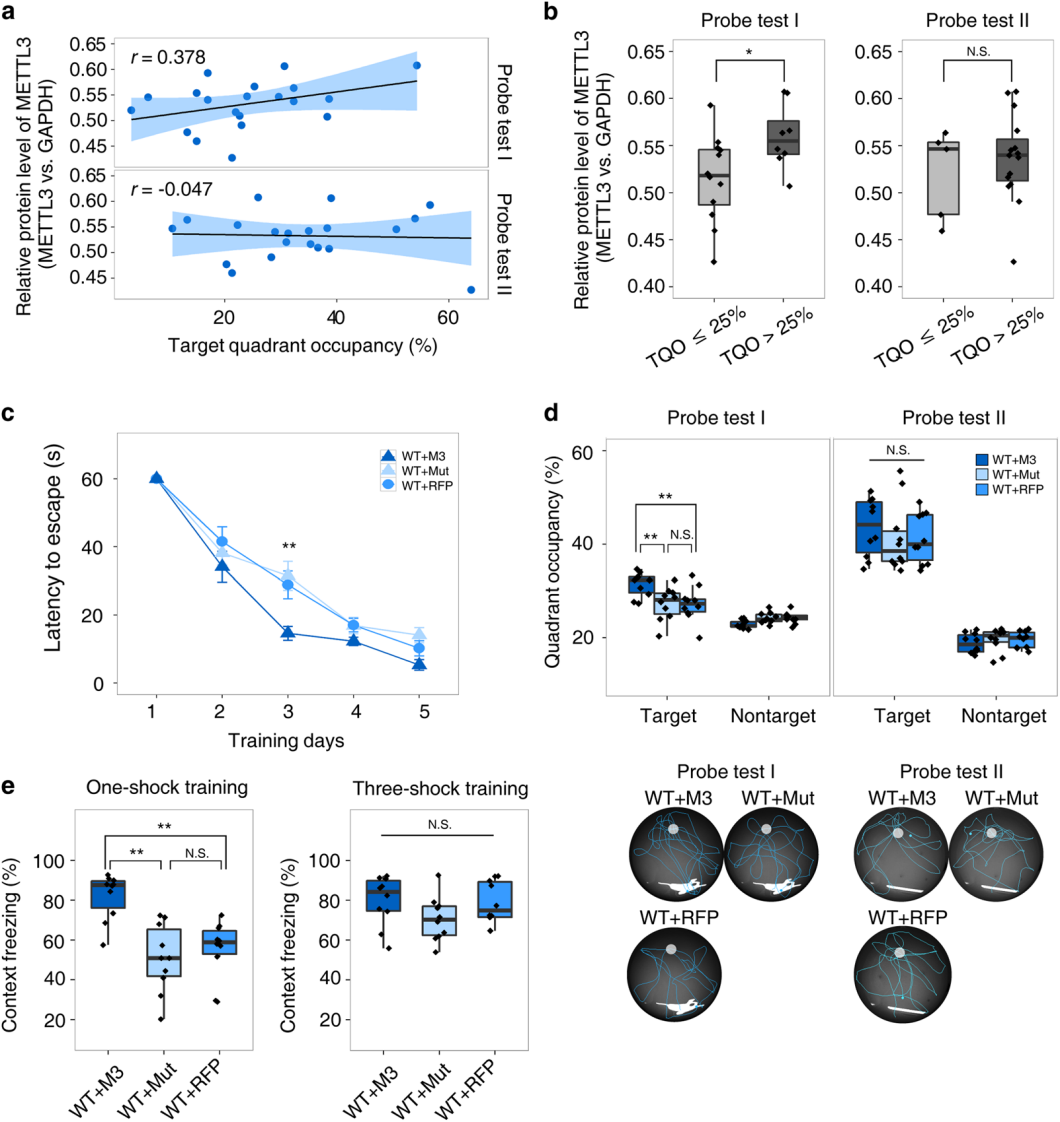
Overexpression of METTL3 enhances long-term memory formation. **a** Correlation between hippocampal METTL3 level with mice performance in two probe tests. *n* *=* 20 mice. *r*, Pearson correlation coefficient. **b** Group difference of MTTL3 abundance between mice with target quadrant occupancy (TQO) < 25% (unlearned) and mice with target quadrant occupancy (TQO) > 25% (learned). **c** Morris water maze test. Group difference was measured by two-way repeated ANOVA, *P* < 0.01. **d** Probe tests of (**c**). **e** Fear conditioning test showing the outperformance learning efficacy of WT + M3 mice vs. WT + Mut and WT + RFP mice in one-shock training but not three-shock training. Student’s *t*-test, *P < 0.05, **P < 0.01, ***P *<* 0.001. N.S., not significant, (**b**) n = 3 replicates, (**c**) to (**e**) *n* *=* 10 mice per group

The above findings suggest that hippocampal METTL3 abundance among individuals may account for the variance of their spatial learning efficacy. Indeed, a moderate positive correlation (*r* = 0.378) between basal hippocampal METTL3 protein abundance and learning efficacy was detected in wildtype mice (8-week-old male) in Morris water maze test (Fig. 6a). Mice with more METTL3 tended to spend more time in the target quadrant in the first probe test, but such correlation disappeared in probe test II after adequate training (Fig. 6b). To further characterize the relationship between METTL3 abundance and learning efficacy, we bilaterally injected AAV2/DJ virus carrying wildtype *Mettl3*, methyltransferase domain-mutated *Mettl3*, or RFP into the dorsal hippocampus of wildtype mice (WT + M3, WT + Mut, and WT + RFP, respectively) (Supplementary Fig. S8). As expected, overexpressing *Mettl3* significantly improved the learning efficacy of mice in both Morris water maze test and one-shock contextual fear conditioning test, but overexpressing *Mettl3* with mutated methyltransferase domain had no effect (Fig. 6c–e), further confirming that METTL3 functions through modulating m^6^A formation. Again, after adequate training (5 days of water maze training and three-shock fear conditioning training), no behavioral difference was detected among *Mettl3* overexpression and other groups (Fig. 6c–e).

## Discussion

Collectively, our work demonstrated that METTL3 enhances hippocampus-dependent long-term memory, likely via promoting the translation efficacy of activity-induced IEGs. Although several previous studies have shown that defects in some m^6^A-related proteins were accompanied with memory impair,^7,8,25,26^ whether those correlations indeed depend on m^6^A remains to be characterized. Here, we have proven that METTL3-mediated long-term memory formation relies specifically on the m^6^A methyltransferase function of METTL3, therefore providing direct evidence for the crucial roles of RNA m^6^A modification in memory consolidation. We found that knocking out *Mettl3* in adult mouse hippocampus does not alter brain anatomical features or short-term memory-related electrophysiological activities, such phenomena should be distinguished from developmental stage studies, in which depletion of *Mettl3* causes severe defects in whole brain.^27–29^ Genes modified by m^6^A at different post-training points shown functional preferences in accordance with the known training-induced physiological processes, indicating that m^6^A modification can response rapidly after training, thus is capable to serve as the mediator between short-term memory and long-term memory.^30^

Many studies have proven that the transcription of IEGs can be robustly and rapidly induced by experience-triggered neuronal activity, hence IEGs are believed to be crucial to link individual experience with long-term memory.^4,5^ We found that the synthesis of IEG proteins is METTL3-dependent. The amount of IEG proteins in *Mettl3* cKO mice produced after training was significantly less than those produced in the CTRL mice, which is in concert with previous reports that insufficient production of IEGs in mice lead to dysfunctions of synaptic plasticity and/or long-term meory.^4,5,20–22^ Thus we reasoned that the insufficient IEG protein production may be one of the causal factors for the long-term memory consolidation defects of *Mettl3* cKO mice. Such process may also be mediated by mRNA translation related m^6^A-binding proteins, such as YTHDF1^24^ and YTHDF3.^31,32^

**Fig. 7:**
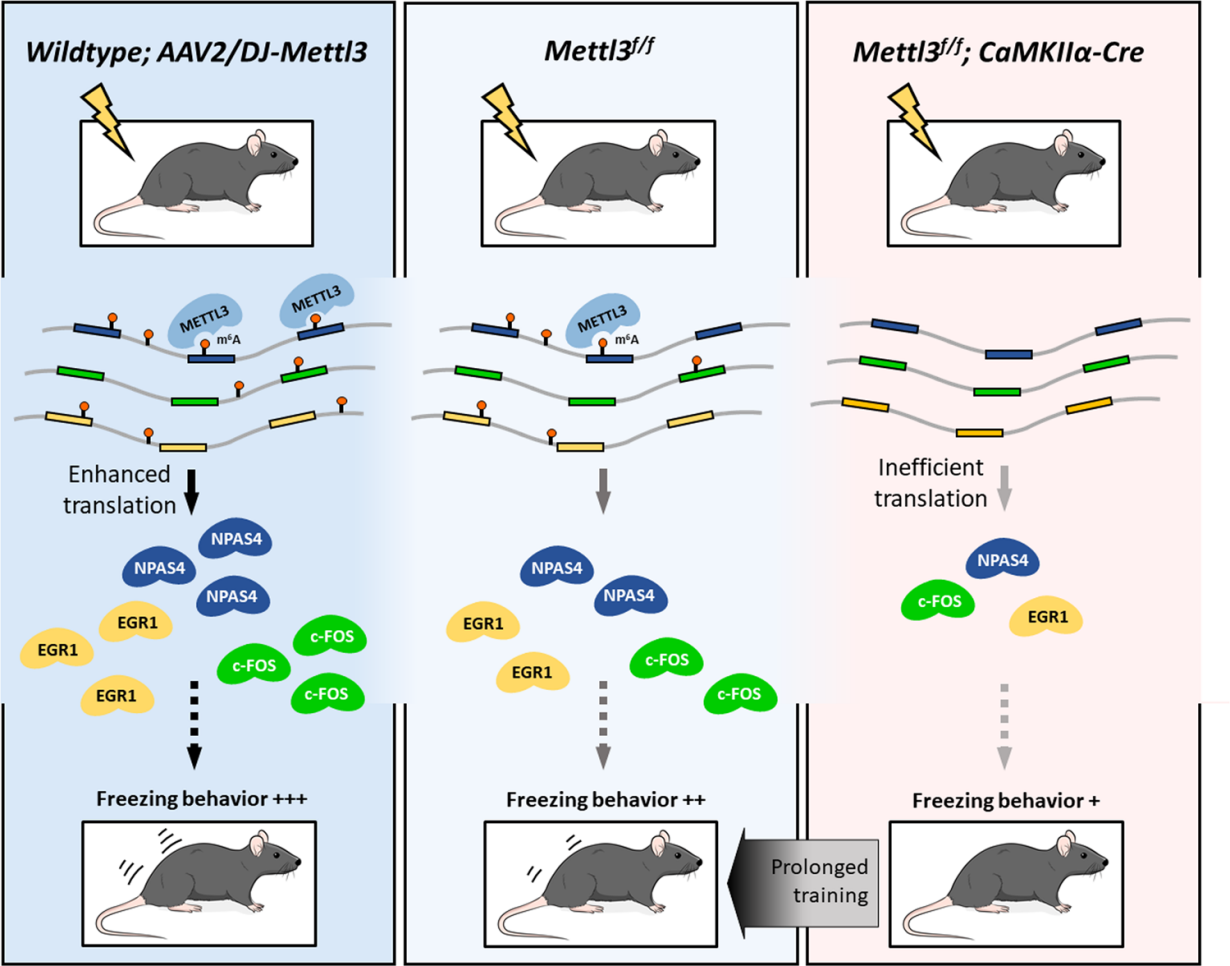
Proposed model: METTL3-mediated m^6^A modification regulates long-term memory consolidation, likely via promoting the translation efficacy of immediate-early genes in mouse hippocampus. Prolonged training compensates m^6^A-deficiency-induced learning defects, and overexpression of METTL3 enhances learning efficacy.

Intriguingly, although the absence of *Mettl3*/m^6^A results in reduced learning efficacy, equal training outcomes can still be achieved after prolonged water maze training or overdose electric shocks, suggesting a beneficial but not indispensable role of m^6^A in regulating memory consolidation (Fig. 7). Such conclusion is further supported by the expression evidence of IEGs, as induction of IEGs after training can still be detected in cKO mice at both the mRNA and protein levels, but much weaker at the protein level in cKO mice than in the CTRL ones. Thus, repetitive induction of IEG proteins at a reduced magnitudes may be able to achieve the required synaptic consolidation effects. The correlation of METTL3 abundance with mouse learning ability suggests that difference in RNA m^6^A induction in hippocampus may be partially responsible for the individual variation in memory formation efficacy, and medicines enhancing *Mettl3* expression or m^6^A formation may improve learning ability and slow down ageing- or disease-related memory loss.

## Materials and Methods

### Animals

All mice were born and raised in a temperature-controlled (22 ± 1 °C) room within ventilated cages inside the specific-pathogen-free barrier, with a 12-hour light/dark lighting cycle (7:30 to 19:30 light hours) and ~ 50% humidity. Pups were kept with their dams after born and weaned at postnatal day 21, then group housed by sex with 4 to 5 mice per cage and *ad libitum* access to food and water. All wildtype mice used in this study were in C56BL/6J genetic background and bought from Vital River Laboratory (Beijing). To generate *Mettl3* conditional knockout mice, *Mettl3*^*flox/flox*^ mice, previously characterized,^27^ were first crossed with *CaMKIIα-Cre* mice (Jax #005359) to generate heterozygous mice (*Mettl3*^*flox/+*^*; CaMKIIα-Cre*), the heterozygous mice were then crossed with either heterozygous or *Mettl3*^*flox/flox*^ mice to generate cKO mice (*Mettl3*^*flox/flox*^*; CaMKIIα-Cre*) and littermate CTRL mice (*Mettl3*^*flox/flox*^). The genotype of each mouse was determined by the genomic DNA extracted from tail tip tissue. All experiments followed the guidelines of the Animal Care and Use Committee of the Institute of Genetics and Developmental Biology, Chinese Academy of Sciences (AT2017022).

### General conditions of behavioral tests

All behavioral tests were carried out on male mice at 8 to 12 weeks of age. Each test was conducted at fixed day time (between 8:30 m to 18:30 pm) on each training day. Animals were handled 2 min for 3 days and transferred to the testing room 24 h prior to the behavioral tests, and those participated in multiple tests were allowed to rest for at least 3 days between two tests. Unless otherwise indicated, the apparatuses were cleaned with 75% ethanol after testing each mouse to prevent any bias caused by olfactory cues. All behavioral tests were carried out with the presence of two researchers blinded to the genotype.

### Elevated-plus maze test

The elevated-plus maze apparatus consists of two opposite open arms (10 × 35 cm) and two opposite closed arms (10 × 35 × 15 cm). The arms are connected to a central square (10 × 10 cm) and installed onto a high rack (60 cm above the ground). The maze is made of opaque plastic in blue color. Each mouse was gently placed in the central zone of the maze facing one of the open arms, then recorded by an overhanging video camera for 10 min. The time spent by each mouse in different arms was analyzed by EthoVision XT 13 (Noduls).

### Open field test

Open field box (40 × 40 × 35 cm) was made of opaque plastic in white color. Each mouse was gently placed in the central zone of the box and allowed to explore freely for 10 min, and recorded by an overhanging video camera. The total move distance and time spent in the center of each mouse were calculated by EthoVision XT 13 (Noduls).

### Rotarod test

Mice (4 per group) were gently placed on a rotating rod (UGO Basile) with an initial speed of 4 rpm for 30 s, then accelerated from 4 rpm to 50 rpm in 5 min. The latency of each mouse falling off the rod was recorded by an electrical relay below the rod. Each mouse was tested 3 times with 3 h break between each trail.

### Novel object recognition test

The novel object recognition test was carried out using the same apparatus as the open field test. Mice were placed into the open field box to habituate for 10 min on the first day. On the second day, each mouse was gently placed in the center of the box, with two objects (a T-25 flask filled with red ink and a 50 ml centrifuge tube filled with water) located in the center zone, and allowed to explore freely for 10 min, then sent back to their home cage. After 30 min, the mice were placed back to the box (with one object changed to a 10 cm tall vase) again for 5 min for retention test. The behavior of each mouse was recorded by EhtoVision XT 13. Frequencies (*f*) investigating each object were manually scored by two experienced researchers. Discrimination index was calculated as (*f*_novel_ - *f*_famliar_) / (*f*_novel_ + *f*_familiar_) ×100%

### Morris water maze test

Morris water maze test was carried out in a circular tank filled with water (120 cm in diameter and 30 cm in depth, made opaque by adding titanium dioxide, maintained at 21 ± 1 °C) in a room with fixed environment. A circular platform (9 cm in diameter) was submerged below the water surface at the center of the target quadrant. For each trial, mouse was gently placed into the water facing the tank wall within one of the four quadrants and allowed to swim for a maximum of 60 s to locate the hidden platform. The releasing quadrant was randomly changed every trial. Mice failed to find the platform were guided toward it with a long metal stick and allowed to stay on the platform for 5 s (testing association between METTL3 protein abundance with learning) or 30 s (all other experiments). Mice were trained twice per day with an interval of 6 h (starting at 8:00 am and 14:00 pm, respectively). During the probe tests, the platform was removed, and mice were allowed to swim for 60 s. The first probe test was carried out on day 4 prior to day 4′s training (16 h after day 3′s last training), and the second probe test was carried out on day 6, 24 h after day 5′s last training. The swimming path and time spent in each quadrant were recorded and analyzed by EthoVision XT 13 (Noduls).

### Contextual fear conditioning test

Mice were gently put into the conditioning box (Panlab, Harvard Apparatus) individually and allowed to explore for 2 min, followed by either one mild electric foot shock (0.8 mA for 2 s) or three mild foot electric shocks (0.8 mA for 2 s each with 60 s interval). Mice were allowed to stay in the conditioning box for another 60 s, then returned to their home cage. Thirty minutes (short-term test) or 24 h later (long-term test), mice were put back to the conditioning box for 5 min. Freezing behavior was recorded and analyzed by PACKWIN 2.0.5 software (Panlab, Harvard Apparatus).

### Immunohistochemistry

Fresh or perfused brain samples were drop-fixed within 4% paraformaldehyde (PFA) in 1× PBS at 4 °C for 48 h, then washed by 1× PBS twice, cryoprotected with 30% sucrose, frozen in Tissue Freezing Medium (TFM, General Data), and sectioned (25–35 µm thick) with a cryostat (Leica). Sections were permeabilized and blocked by blocking buffer containing 0.2% Triton X-100 and 2% bovine serum in 1× PBS for 30 min at room temperature. Sections were then incubated with primary antibodies diluted with blocking buffer at 4 °C overnight, and secondary antibodies diluted with blocking buffer for 2 h at room temperature. Nuclei were stained by DAPI with mounting medium (Vectorlabs, #H-1200). Antibodies used for immunofluorescent labeling were as follows: anti-METTL3 (1:200 dilution, Abcam, ab195352), anti-CUX1 (10 μg/ml, Abcam, ab54583), anti-EGR1 (1:500 dilution, Invitrogen, MA515008), goat-anti-rabbit Alexa Fluor 594 (1:500, Abcam, ab150080), donkey-anti-rabbit Alexa Fluro 488 (1:500, Abcam, ab150073) and goat-anti-mouse Alexa Fluor 488 (1:500, Abcam, ab150113). Images were acquired using Leica SP8 confocal microscope.

### Hematoxylin-eosin (HE) staining

Brain tissues (fixed in 4% PFA at 4 °C for at least 48 h) were automatically dehydrated by Tissue-Tek VIP5Jr (Sakura) and embedded in paraffin (56–58 °C) with Tissue-Tek TEC 5 Tissue Embedding Console System (Sakura). Brain paraffin blocks were cut into 8-μm thick sections by a manual rotary microtome (Leica, RM2235), and the sections were affixed onto poly-D-lysine coated microscope slides (CITOGLAS, 10127105 P). For hematoxylin-eosin staining, sections were first dewaxed in xylene (2 times, 10 min each) and rehydrated (sequentially 100% alcohol 5 min, 95% alcohol 3 min, 70% alcohol 3 min, and rinsed in distilled water), then automatically stained by Tissue-Tek Multiple Slide Stainer (Sukara, DRS 2000). The stained sections were dehydrated in 100% alcohol (3 times, 5 min each), cleared in xylene (3 times, 5 min each) and mounted with neutral balsam (Solarbio, G8590). Whole-slide images were obtained by scanning the slides with NanoZoomer RS scanner (Hamamatsu).

### Nissl staining

Nissl staining was performed using a staining kit (Coolabler, DZSL0135) following the manufacture’s instruction. Briefly, paraffin-embedded sections were dewaxed and hydrated following the same protocol as described in HE staining. Sections were then stained in cresyl violet staining solution in a 56 °C incubator for 1 h, washed by distilled water and then submerged into the differentiation solution provided by the kit until the background became clear. Stained sections were next dehydrated in 100% alcohol (3 times, 5 min each), cleared in xylene (2 times, 5 min each), and mounted with neutral balsam (Solarbio, G8590). Whole-slide images were obtained by scanning the slides with NanoZoomer RS scanner (Hamamatsu).

### TUNEL staining

TUNEL staining was performed using *in situ* cell death detection kit (Roche, 11684809910) following the manufacture’s instruction. Briefly, cryopreserved tissue sections (prepared following procedures described in the immunohistochemistry experiment) were post-fixed with 4% PFA in 1× PBS for 20 min at room temperature, then washed with 1× PBS for 30 min, and permeabilized with 0.1% Triton X-100 in 0.1% sodium citrate for 2 min at 4 °C. Each tissue section was stained with 50 μl label solution plus 50 μl enzyme solution (all provided by the kit) in a humidified 37 °C incubator for 1 h, rinsed in 1× PBS for 3 times, then counter stained with DAPI following procedures described in the immunochemistry experiment. Slides were imaged using a Leica fluorescence microscope (DMI 3000B). Positive and negative control staining samples were performed following the manufacture’s instruction to confirm the validity of the staining results (data not shown).

### Electrophysiological recordings

Mice (8 weeks) were deeply anesthetized by pentobarbital sodium (2%, 0.3 ml/100 g) and decapitated. The brains were rapidly removed and kept in ice-cold artificial cerebrospinal fluid (ACSF, in mM: 124 NaCl, 2.5 KCl, 1.2 NaH_2_PO_4_, 24 NaHCO_3_, 12.5 D-glucose, 2 CaCl_2_, and 1.5 MgSO_4_, saturated with 95 % O_2_ and 5 % CO_2_, pH adjusted to 7.3, and osmolarity adjusted to ~ 295 mOsm with sucrose). Hippocampal acute brain slices (300 µm thick) were prepared with a vibratome (Leica) which was filled with ice-cold ACSF and incubated within oxygenated ACSF at room temperature for 1 h. Then individual brain slices were transferred to a recording chamber bubbled with oxygenated ACSF at 31 ± 1 °C (2 ml/min perfusion rate). CA1 pyramidal neurons were visually targeted by using an Olympus microscope (Olympus BX50-WI). Patch pipettes with 4–6 MΩ resistance were pulled from 110 mm borosilicate glass capillaries (Sutter Instrument). The internal solution used was (in mM): 140 K-gluconate, 2 MgCl_2_, 8 KCl, 10 HEPES, 0.2 Na-GTP, 2 Na_2_-ATP (pH = 7.3). Recordings and analysis were performed by Axopatch 700B amplifier (AXON), Digidata 1440 A (Molecular Devices) and pCLAMP 10.6 software (Molecular Devices). The series and input resistances were monitored throughout each experiment, cells simultaneously satisfying the following requirements (high seal resistance > 1 GΩ, series resistance below 25 MΩ, series resistance and input resistance changed less than 15%) were included for further analysis.

Miniature excitatory postsynaptic current (mEPSC) was recorded at −70 mV by holding the cells under whole-cell voltage-clamp mode. To isolate AMPA receptor-mediated mEPSCs, recordings were obtained in ACSF with 1 µM TTX and 100 µM PTX. Each cell was recorded for at least 5 min.

Action potential (AP) was recorded under whole-cell current-clamp mode and was evoked by a series of depolarizing current pulses (500 ms) from −60 to 500 pA with a 20 pA step increment. A single 500 pA current (500 ms) was injected to measure the inter-spike intervals, and 3 repeated −20 pA currents (800 ms) were injected to measure the fast/slow after-hyperpolarizing potentials (f/sAHPs).

### Short-term plasticity and long-term potentiation measurement

Acute brain slices (380 μm) were prepared as described above and incubated in oxygenated ACSF at room temperature for 1.5 h. Individual slices were transferred to recording chamber bubbled with oxygenated ACSF at 31 ± 1 °C (6 ml/min perfusion rate). Extracellular recording electrodes were filled with ACSF and positioned at the *stratum radiatum* of CA1 area of dorsal hippocampus. Concentric stimulation electrode was placed in *sradiatum* of CA3. Each recording started with measuring the input/output ratio by adjusting the stimulus intensity from 0 to 80 μA with an increment of 5 μA. Paired pulse ratio (PPR) was assessed by applying a succession of paired pulses separated by time intervals of 20 ms, 50 ms, 100 ms, and 200 ms. Waiting for 0.5–1 h, the same brain slice was further recorded for long-term potentiation (LTP). Stimulation intensity was set by eliciting 40% of a maximal response as the baseline level. A stable baseline was achieved for at least 30 min prior to theta-burst stimulation (TBS), after which the LTP was recorded for 1 h.

### Virus preparation

The cDNA of *Mettl3* gene was amplified from mouse, and cloned into the T-vector (Transgen, CB101). The DPPW motif (residues 395–399) of *Mettl3* (wildtype Mettl3), which is important for AdoMet binding, was mutated into APPA (mutated *Mettl3*) by PCR site-directed mutagenesis. The wildtype *Mettl3* and mutated *Mettl3* were subcloned into pAAV2/DJ-CMV-MSC-RFP vector (HANBIO). The pAAV-RC and pHelper were co-transfected with pAAV2/DJ-CMV-wildtype-*Mettl3*-RFP (AAV2/DJ-WT-*Mettl3*), pAAV2/DJ-CMV-mutated-*Mettl3*-RFP (AAV2/DJ-Mut-*Mettl3*) or pAAV2/DJ-CMV-MSC-RFP (AAV2/DJ-RFP) into AAV-293 cells by using LipoFiter transfection reagent (HANBIO) to generate the adeno-associated virus (AAV). Propagated AAV2/DJ in the AAV-293 cells were purified and the titer of virus was measured by plaque assays. The stock solutions of AAV2/DJ-WT-*Mettl3*, AAV2/DJ-Mut-*Mettl3* and AAV2/DJ-RFP were 1.0–1.2 × 10^12^ plaque formation unit (PFU)/ml, respectively. Primers are listed in Supplementary Table S2.

### Stereotaxic injection

Adult mice (8 weeks) were anesthetized with isoflurane and placed in a stereotaxic apparatus (RWD). Viruses were delivered via a Hamilton syringe at a rate of 0.1 µl per minute, and needles were kept still for an additional 1 min before withdrawing. One microliter AAV2/DJ viruses carrying wildtype *Mettl3*, mutated *Mettl3* or RFP (all 1.0–1.2 × 10^12^ PFU/ml), respectively, were bilaterally injected into the dorsal hippocampus (relative to bregma: AP = −1.9 mm, ML = ± 1.2 mm, DV = − 1.3 mm). After the surgery, mice were kept on a warm pad for a short period of recovery, then returned to their home cage and monitored for 24 h. Mice were housed for 2 weeks after surgery before behavioral tests.

### RNA isolation

TRNzol Universal (TIANGEN, DP424) was used to extract total RNA from cells or hippocampal tissue. RNA concentration was measured using NanoPhotometer P330 (Implen), and only samples with OD 260/280 nm ratio of ~2.0 were used for subsequent experiments. The integrity of RNA was tested by agarose gel electrophoresis of total RNA, and only RNA samples with 28 S and 18 S ribosomal RNA gel bands at an approximate ratio of 2:1 were used for further study.

### Methylated RNA immunoprecipitation

Methylated RNA immunoprecipitation (MeRIP) was performed using Epimark *N*^6^-Methyladenosine Enrichment Kit (NEB, E1610S) on fear conditioning trained hippocampus tissues (30 min and 1 h pooled samples). Briefly, 2 µl m^6^A antibody was attached to protein G magnetic beads (NEB, S1430). Then, 100 µg total RNA with m^6^A control RNA (Gaussia luciferase, GLuc) and unmodified control RNA (Cypridina luciferase, CLuc) was incubated with beads at 4 °C for 1 h. The beads were separately washed twice with reaction buffer (150 mM NaCl, 10 mM Tris-HCl, pH 7.5, 0.1% NP-40 in nuclease free H_2_O), low salt reaction buffer (50 mM NaCl, 10 mM Tris-HCl, pH 7.5, 0.1% NP-40 in nuclease free H_2_O), and high salt reaction buffer (500 mM NaCl, 10 mM Tris-HCl, pH 7.5, 0.1% NP-40 in nuclease free H_2_O). The enriched m^6^A-containing RNA was purified by phenol–chloroform extraction.

### qRT-PCR

The extracted RNA was treated with DNase I (ThermoFisher Scientific, EN0525) and reversely transcribed into cDNA by reverse transcriptase (ThermoFisher Scientific, EP0441). SYBR Green PCR Master Mix (Toyobo, QPK-201) was used in qRT-PCR experiments. The 2^-△△Ct^ method was performed to calculate relative expression. Primers are listed in Supplementary Table S2.

### m^6^A dot blot assay

The m^6^A dot blot assay was carried out on a Bio-Dot Apparatus (Bio-Rad, 84BR-31918) as previously described^33^ with minor modification. Briefly, total RNA isolated from rapid frozen (by liquid nitrogen) hippocampus tissue (8 weeks male) was quantified using a NanoPhotometer P330 (Implen), 500 ng total RNA from each sample was spotted onto a positive-charged nylon-based membrane (GEHealthcare, RPN303B) unless otherwise indicated. RNA samples were then blocked by 5% skim milk (Amresco, M203–10G) dissolved in blocking buffer (LI-COR, 927–50000) at room temperature for 2 h, and incubated with primary antibody anti-m^6^A (1:3000 in blocking buffer, Abcam, ab151230) at 4 °C overnight. RNA samples were next washed by 1× TBST (3 × 5 min, CWBIO, CW00435), incubated with secondary antibody IRDye 800CW (1:5000 in blocking buffer, Odyssey, 926–32211) at room temperature for 2 h, and washed again with 1× TBST (2 × 5 min). Images were obtained from ODYSSEY CLx (LI-COR) and analyzed by ImageJ (v1.51 K).

### miCLIP-SMARTer-m^6^A-seq

Small scale single-base resolution m^6^A methylome detection was carried out following procedures modified from a previously report.^13^ Briefly, 100 ng mRNAs were isolated from 5 mice (8 weeks male) hippocampus using Dynabeads mRNA Purification Kit (Life Technologies, 61006) and fragmented to ~100 nt using fragmentation reagent (Life Technologies, AM8740), then incubated with 5 µg anti-m^6^A antibody (Abcam, ab151230) in 450 µl immunoprecipitation buffer (50 mM Tris, 100 mM NaCl, 0.05% NP-40, adjusted to pH 7.4) under gentle rotation at 4 °C for 2 h. The mixture was then transferred into a clear flat-bottom 96-well plate (Corning) on ice, and irradiated three times with 0.15 J/cm^−2^ at 254 nm in a CL-1000 Ultraviolet Crosslinker (UVP). After irradiation, the mixture was collected and incubated with 50 µl pre-washed Dynabeads Protein A (Life Technologies, 1001D) at 4 °C for 2 h. After extensive washing by high-salt buffer (2 times, 50 mM Tris, 1 M NaCl, 1 mM EDTA, 1% NP-40, 0.1% SDS, adjusted to pH 7.4) and immunoprecipitation buffer (2 times), and dephosphorylation with T4 PNK (NEB, M0201L) at 37 °C for 20 min on beads. The RNA was eluted from the beads by proteinase K (Sigma, P2308) treatment at 55 °C for 1 h, followed by phenol-chloroform extraction and ethanol precipitation. Purified RNA was subjected to library construction using SMARTer smRNA-Seq Kit for Illumina (Clontech Laboratories, 635030) according to the manufacturer’s instructions and sequenced on Illumina HiSeq X Ten platform.

### RNA-seq

RNA sequencing samples were prepared according to the instruction of TruSeq RNA Sample Prep Kit (Illumina, FC-122–1001). Briefly, total RNAs (~5 µg) were extracted from rapid frozen mice hippocampus tissue and used to generate cDNA libraries. All samples were sequenced on Illumina HiSeq X Ten platform. Two replicates were sequenced (each replicate represents one mouse) for each condition.

### Synaptosomal fractionation, protein extraction and western blot

Synaptosomes from mice fresh hippocampus were isolated by Syn-PER Reagent (ThermoFisher Scientific, 87793). Total proteins from either mice hippocampus or primary cortical neurons were extracted by N-PER Neuronal Protein Extraction Reagent (ThermoFisher Scientific, 87792). Pierce Coomassie Protein Assay Kit (ThermoFisher Scientific, 23200) was used to calculate the protein concentration. Protein fraction (~50 µg) was separated by 10% SDS-PAGE and analyzed by immunoblotting with corresponding antibodies, anti-GluN2A (1:2000, Abcam, ab124913), anti-GluN1 (1:2000, Abcam, ab109182), anti-HOMER1 (1:3000, Abcam, ab184955), anti-GluN2B (1:5000, Abcam, ab81271), anti-GluR1 (1:2000, Abcam, ab109450), anti-GluR2 (1:2000, Abcam, ab133477), anti-SHANK1 (1:300, Proteintech, 55059-1-AP), anti-PSD95 (1:3000, Abcam, ab76115), anti-CAMK2A (1:3000, Abcam, ab52476), anti-YTHDF1 (1:1000, Proteintech, 17479-1-AP) and anti-eIF2α (1:1000, Abcam, ab169528), anti-METTL3 (1:1000, Abcam, ab195352), anti-beta TUBULIN (1:2000, Abcam, ab108342), anti-GAPDH (1:7500, Proteintech, 60004-1-AP), anti-c-FOS (1:1000, Abcam, ab214672), anti-EGR1 (1:1000, ThermoFisher Scientific, MA5-15008), anti-NPAS4 (1:500, Abcam, ab109984), anti-ARC (1:1000, Abcam, ab183183), anti-NR4A1 (1:1000, Abcam, ab109180). IRDye secondary antibodies were used for protein detection by the LI-COR Odyssey imaging systems (ODYSSEY CLx, LI-COR). The relative protein levels were analyzed by ImageJ (v1.51K).

### Analysis of miCLIP-m^6^A-seq data

miCLIP-seq data (paired-end) were analyzed as previously described.^13,14^ Briefly, adaptor sequences were trimmed by Cutadapt (v1.7.1) with parameters: -q 5 -O 5 -m 20. Forward reads were demultiplexed by fastq2collapse.pl (CTK Tool Kit, v1.0.9) and the reverse reads were first transformed to reverse complementary sequences using fastx_reverse_complement (FASTX Toolkit, v0.0.13) then processed in the same way. Next, random barcodes were striped by stripBarcode.pl (CTK Tool Kit, v1.0.9) and attached to the read headers to facilitate downstream CIMS analysis, then we pull the forward and transformed reverse reads of each sample into a single file and align them to the reference genome (mm10, UCSC Genome Browser) using BWA (v0.7.12-r1039) with parameters: -n 0.06 -q 35. Cross-linking-induced mutation sites (CIMSs) were identified using CTK Tool Kit (v1.0.9): uniquely aligned reads were selected using parseAlignment.pl (--map-qual 1 --min-len 18) and PCR duplicates were collapsed using tag2collapse.pl (-EM 30 --seq-error-model alignment). Mutation sites (insertions, deletions and substitutions) were then identified using joinWrapper.py, and CIMS C → T transitions were specified using CIMS.pl (-n 10). Only CIMS sites with transition number ≥ 2 (*m* ≥ 2) and transition to total coverage ratio between 1 and 50% (0.01 ≤ *m*/*k* ≤ 0.5) were selected for further analysis. Adenosines positioned 5’ adjacent to CIMS sites were identified as m^6^A sites and annotated by bed2annotation.pl (-dbkey mm10). Metagene distribution was analyzed using metaPlotR, and motif enrichment analysis was performed using findMotifs.pl (Homer v4.8).

### Analysis of RNA-seq data

Paired-end, adapter-clean reads were first aligned to the reference genome (mm10, USCS Genome Browser) using Tophat2 (v2.1.1) with default parameters. Cufflinks (v2.2.1) was used to assemble uniquely mapped reads into transcripts and estimate respective abundance (FPKM) with default parameters. Differentially expressed genes between samples were identified by using Cuffdiff (2.2.1) with fold change ≥ 2 and q-value ≤ 0.05 as thresholds. Gene ontology (biological process) enrichment analysis was performed using Metascape online service (*metascape.org/gp/index.html#/main/step1*).

### Cell culture

Primary neurons were isolated from the cortical tissues of E18.5 *Mettl3*^*f/f*^ embryos without discrimination of sex. Briefly, brain cortical tissues were dissociated with Neural Tissue Dissociation Kit (Miltenyi, 130092628) following the manufacture’s instruction. Neurons were plated onto Matrigel (Corning, 354227) pre-coated plates (Coring) at a density of 5 × 10^5^ cells/cm,^2^ and cultured with Neurobasal (Gibco, 21103049) supplemented with 2% B-27 (Gibco, 17504044), 1% GlutaMAX (Gibco, 35050061), 1% non-essential amino acids solution (Gibco, 11140050), and 1% penicillin-streptomycin-neomycin antibiotic mixture (Gibco, 15640055). Cells were maintained under 37 °C and 5% CO_2_ conditions, and the culture medium was half-changed every two days. For *Mettl3* knockout and rescue experiment, neurons were first infected with adenovirus expressing Cre-GFP (HANBIO, HBAD1016) or GFP (HANBIO, HBAD1009) at DIV 3 (3 days *in vitro*) to achieve *Mettl3* knockout, and infected with AAV2/DJ expressing wildtype *Mettl3*, mutated *Mettl3*, or RFP at DIV 5. For overexpressing *Mettl3* experiment, neurons were directly infected with AAV2/DJ expressing wildtype *Mettl3*, mutated *Mettl3*, or RFP at DIV 5. Prior to infection, half of the culture medium was collected, and the neurons were incubated with respective viruses overnight. On the next day, cells were washed and changed to culture medium composed of the collected culture medium prior to virus infection (50%), and the fresh culture medium (50%). At DIV 8 to 10, neurons were stimulated with 25 mM KCl (Sigma, P5405) for 1 h in the incubator, then collected for downstream analysis.

### Statistical tests

Results are presented in boxplot (median, 25^th^ and 75^th^ percentiles) with data points plotted inside the box or in bar plots and dot plots as mean ± SEM. Statistical analysis and plot drawing were performed using either Prism GraphPad 5 or R (v3.1.3). Data distribution was presumed to be normal and homoscedastic between groups, but this was not formally tested. Comparison between two groups was analyzed by two-tailed Student’s *t*-test and Pearson correlation, and comparison between three or more groups was analyzed by one-way or two-way ANOVA and Tukey’s Honest Significant Differences (Tukey’s HSD) *post hoc* test unless otherwise indicated. The statistical tests, exact *P* values, sample sizes (n) for each experiment are specified in the figure legend.

### Data availability

All sequencing data generated in this study, including the RNA-seq and miCLIP-m^6^A-seq datasets, are deposited in the Genome Sequence Archive in BIG Data Center (http://bigd.big.ac.cn) under the accession number: CRA001077.

## Electronic supplementary material

Supplementary information, Figure S1

Supplementary information, Figure S2

Supplementary information, Figure S3

Supplementary information, Figure S4

Supplementary information, Figure S5

Supplementary information, Figure S6

Supplementary information, Figure S7

Supplementary information, Figure S8

Supplementary information, Table S1

Supplementary information, Table S2
