# Supplementary material for: METTL3-mediated N6-methyladenosine mRNA modification enhances long-term memory consolidation
Source: Cell Res. 2018 Oct 8;28(11):1050–61. doi: 10.1038/s41422-018-0092-9 (PMC6218447; doi:10.1038/s41422-018-0092-9)
Supplement: Supplementary file 1 — Supplementary information, Figure S1 [file 41422_2018_92_MOESM1_ESM.pdf]

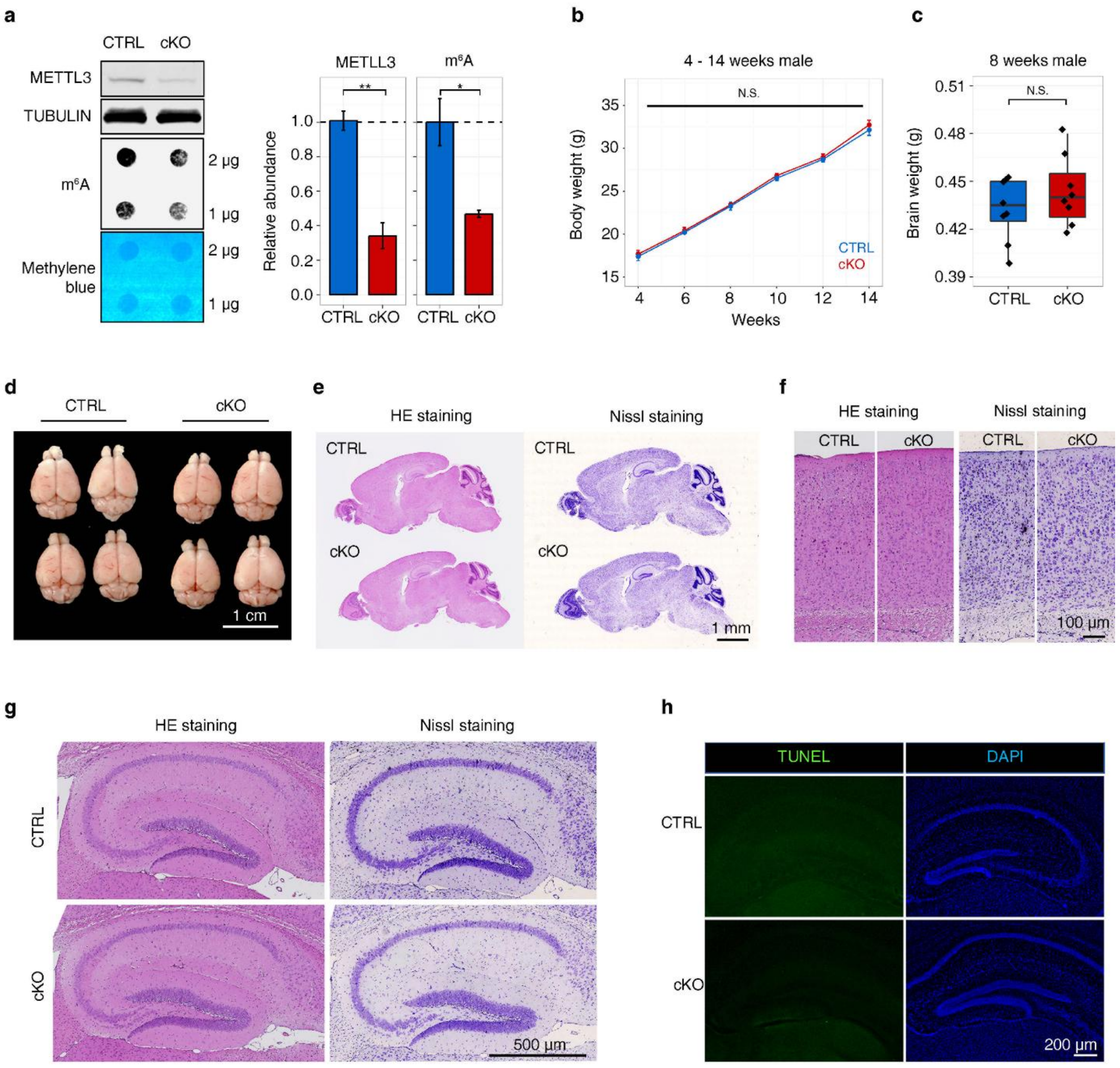

Fig. S1. Characterization of brain gross morphology of *Mettl3* cKO mice.

a CaMKIIα-Cre-mediated KO of *Mettl3* decreases m^6A abundance in hippocampus (n = 3 replicates). cKO mice developed normally into adulthood with normal (**b**) body weight, (**c**) brain weight, (**d** to **g**) brain morphology and (**h**) without observable apoptosis in hippocampus (8 weeks). Student's *t*-test, *$P < 0.05$, **$P < 0.01$, N.S., not significant; (**b**) and (**c**), n = 8 mice per group.
