# Supplementary material for: METTL3-mediated N6-methyladenosine mRNA modification enhances long-term memory consolidation
Source: Cell Res. 2018 Oct 8;28(11):1050–61. doi: 10.1038/s41422-018-0092-9 (PMC6218447; doi:10.1038/s41422-018-0092-9)
Supplement: Supplementary file 2 — Supplementary information, Figure S2 [file 41422_2018_92_MOESM2_ESM.pdf]

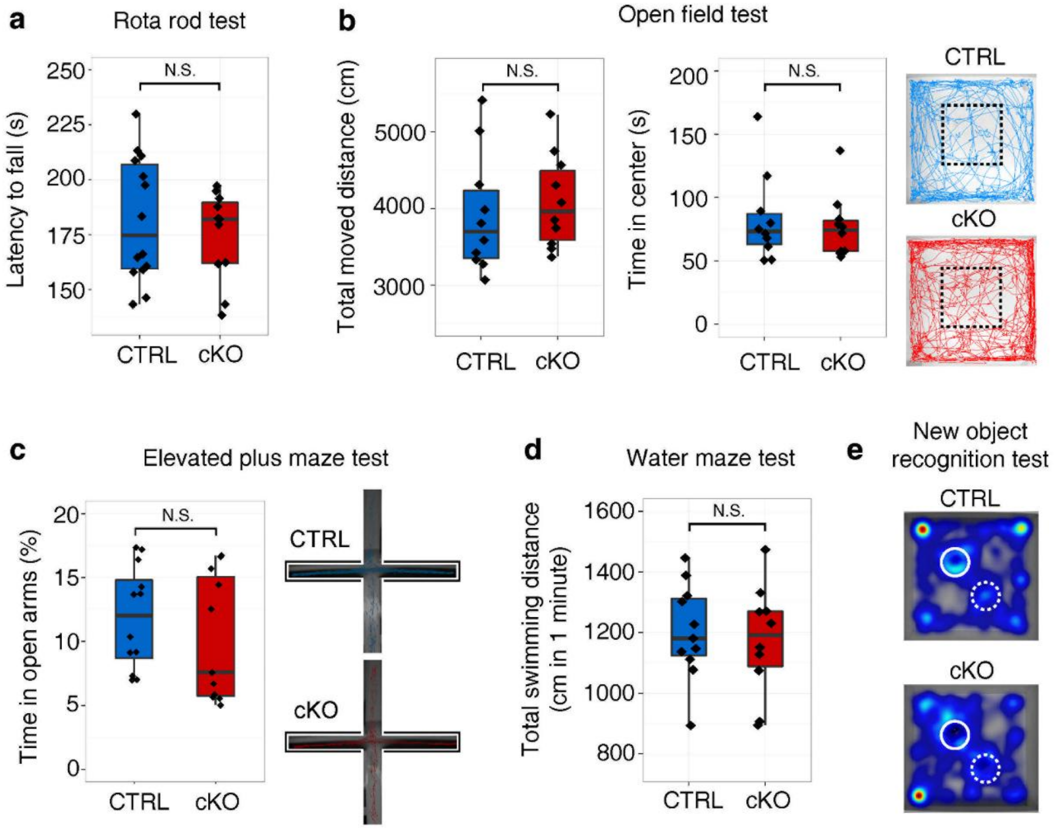

Fig. S2. *Mettl3* cKO mice show no difference in locomotion, exploration and anxiety as compared to CTRL.

a Rotarod test measures animals' motor coordination (CTRL, n = 14 mice; cKO, n = 11 mice). **b** Total moved distance within 10 min in an open field arena (left), duration of mice spent in central zone (middle) and representative moving traces (right) (n = 10 mice per group). **c** Duration of mice spent in open or closed arms (left) and representative traces (right) (CTRL, n = 12 mice; cKO, n = 11 mice). **d** Total swimming distance of mice (CTRL, n = 11 mice; cKO, n = 10 mice). **e** Representative heatmaps showing animals' preference for the novel object (solid circle) over the old object (dashed circle). Student's t-test, N.S., not significant.
