# Supplementary material for: METTL3-mediated N6-methyladenosine mRNA modification enhances long-term memory consolidation
Source: Cell Res. 2018 Oct 8;28(11):1050–61. doi: 10.1038/s41422-018-0092-9 (PMC6218447; doi:10.1038/s41422-018-0092-9)
Supplement: Supplementary file 3 — Supplementary information, Figure S3 [file 41422_2018_92_MOESM3_ESM.pdf]

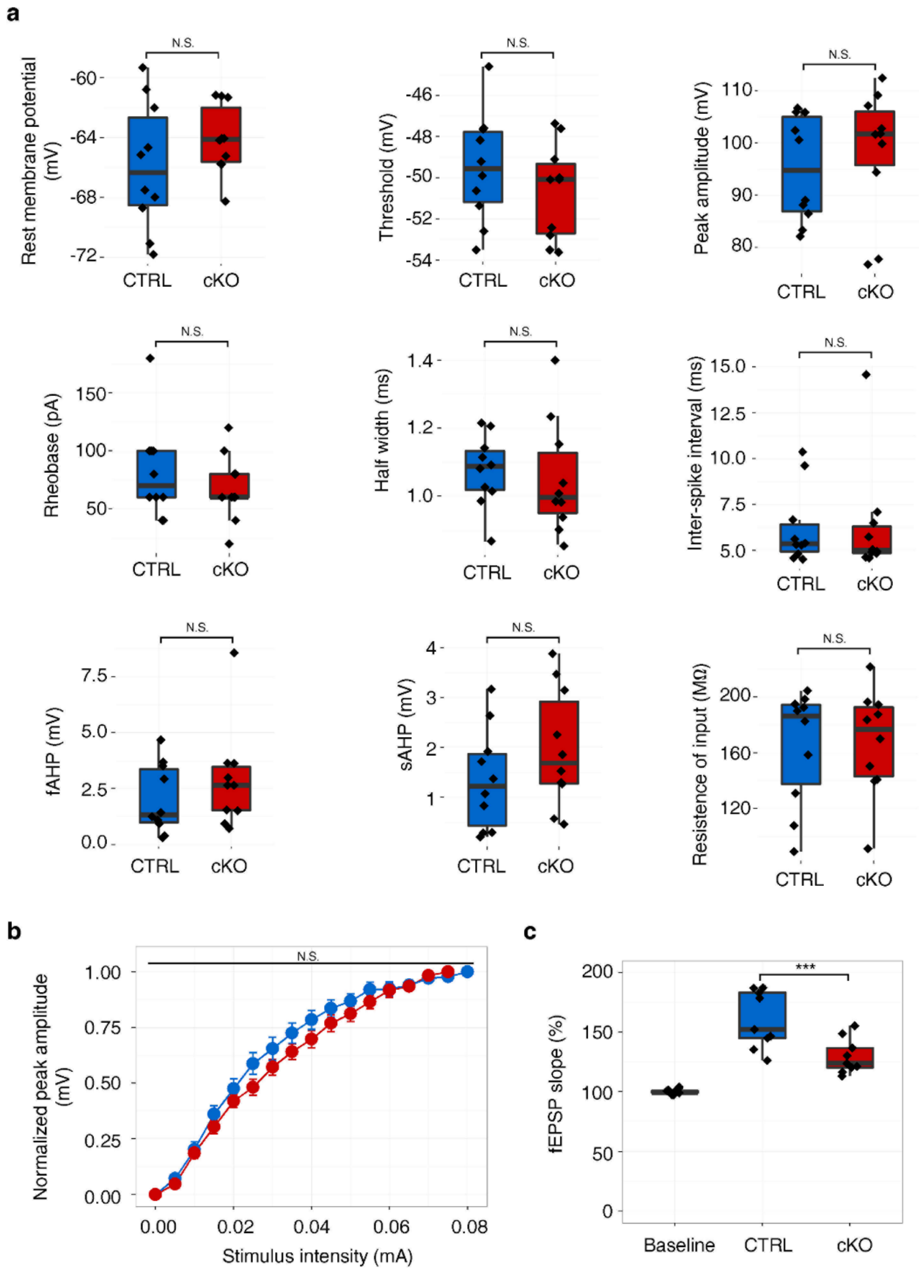

Fig. S3. Characterization of electrophysiological properties of cKO mice.

a Measurements from whole cell recordings of CA1 pyramidal neurons. fAHP, fast after-hyperpolarization; sAHP, slow after-hyperpolarization. **b** Input-output curve of fEPSP in response to different stimulus in CA1 region. **c** Slope of fEPSP during the last 10 min of LTP recording. Baseline is measured during the last 10 min before theta-burst stimulation. (**a**), n = 10 brain slices from 3 mice per group. (**b**) and (**c**), n =9 brain slices from 3 mice per group. Student's t-test, ***P < 0.001, N.S., not significant. CTRL group is marked in blue and cKO group is marked in red.
