# Supplementary material for: METTL3-mediated N6-methyladenosine mRNA modification enhances long-term memory consolidation
Source: Cell Res. 2018 Oct 8;28(11):1050–61. doi: 10.1038/s41422-018-0092-9 (PMC6218447; doi:10.1038/s41422-018-0092-9)
Supplement: Supplementary file 4 — Supplementary information, Figure S4 [file 41422_2018_92_MOESM4_ESM.pdf]

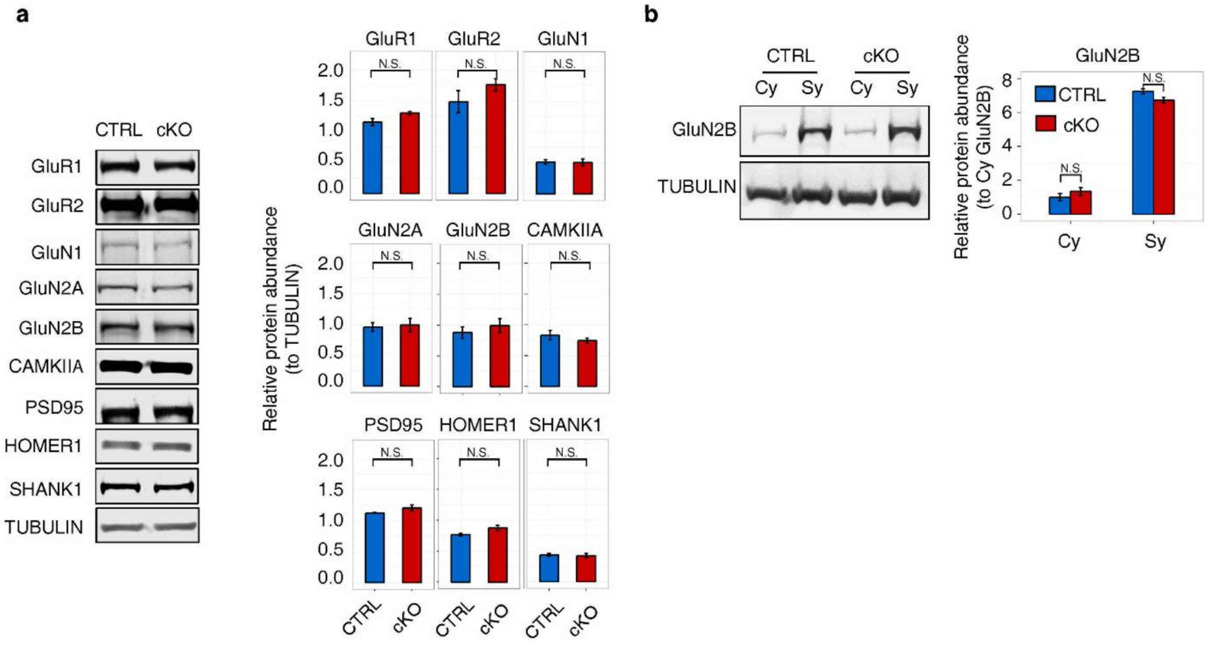

Fig. S4. Characterization of key synaptic proteins at synaptosomal fraction derived from the hippocampus of na ïve mice.

a Western blot (left) and relative grey-level abundance (right, using TUBULIN as the control) show cKO mice have normal abundance of synaptic function-related proteins in hippocampus. **b** Validation of synaptosomal fractionation by blotting GluN2B and TUBULIN in cytosolic and synaptosomal fractions (left) and evaluating the relative protein abundance of GluN2B (right, using cytosolic GluN2B as the control). Cy, cytosol; Sy, synaptosome. Student's *t*-test, N.S., not significant. $n = 3$ replicates.
