# Supplementary material for: METTL3-mediated N6-methyladenosine mRNA modification enhances long-term memory consolidation
Source: Cell Res. 2018 Oct 8;28(11):1050–61. doi: 10.1038/s41422-018-0092-9 (PMC6218447; doi:10.1038/s41422-018-0092-9)
Supplement: Supplementary file 5 — Supplementary information, Figure S5 [file 41422_2018_92_MOESM5_ESM.pdf]

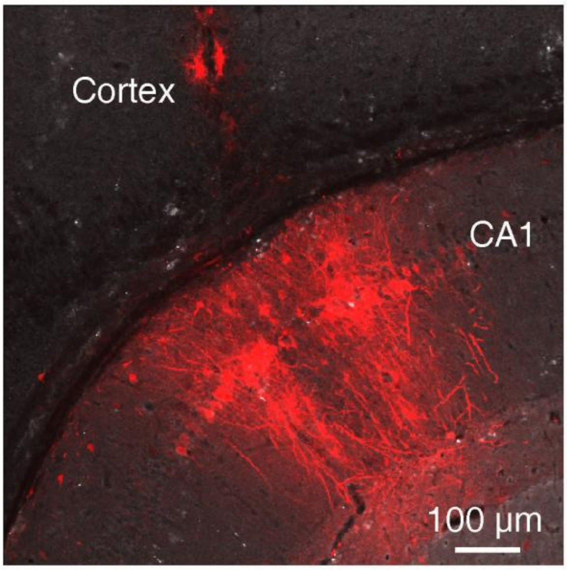

Relative to bregma: AP = −1.9 mm
ML = ±1.2 mm
DV = −1.3 mm

Fig. S5. Representative image of AAV2/DJ virus injection region.
Fluorescent image of brain regions expressing RFP carried by AAV2/DJ viruses after 2 weeks of injection. Cortex and CA1 region of dorsal hippocampus are marked in the image. Coordinates of the injection position are listed under the image.
