# Supplementary material for: METTL3-mediated N6-methyladenosine mRNA modification enhances long-term memory consolidation
Source: Cell Res. 2018 Oct 8;28(11):1050–61. doi: 10.1038/s41422-018-0092-9 (PMC6218447; doi:10.1038/s41422-018-0092-9)
Supplement: Supplementary file 6 — Supplementary information, Figure S6 [file 41422_2018_92_MOESM6_ESM.pdf]

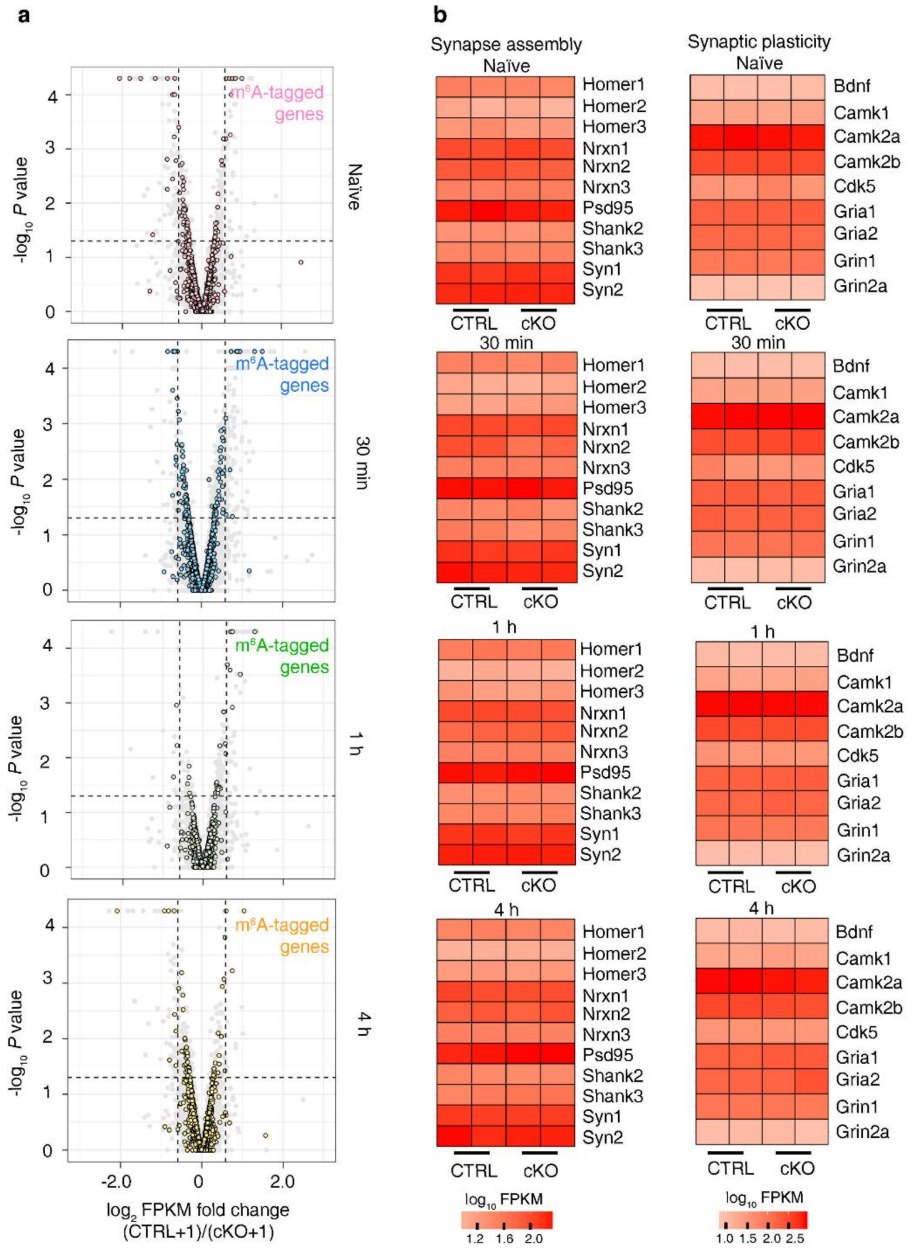


Fig. S6. Analysis of transcriptomic change during early timepoints after training.
**a** Volcano plots show no dramatic transcriptomic abundance change (FPKM) between CTRL and cKO mice at all timepoints. $m^6A$-tagged genes are color-coded, respectively. **b** Expression heatmaps of key genes related to synaptic functions between cKO and CTRL mice at respective timepoints (all genes listed are modified by $m^6A$ except for *Cdk5*, which is not identified from miCLIP-$m^6A$-seq). Two RNA-seq replicates are included in both the CTRL and cKO groups.
