# Supplementary material for: METTL3-mediated N6-methyladenosine mRNA modification enhances long-term memory consolidation
Source: Cell Res. 2018 Oct 8;28(11):1050–61. doi: 10.1038/s41422-018-0092-9 (PMC6218447; doi:10.1038/s41422-018-0092-9)
Supplement: Supplementary file 7 — Supplementary information, Figure S7 [file 41422_2018_92_MOESM7_ESM.pdf]

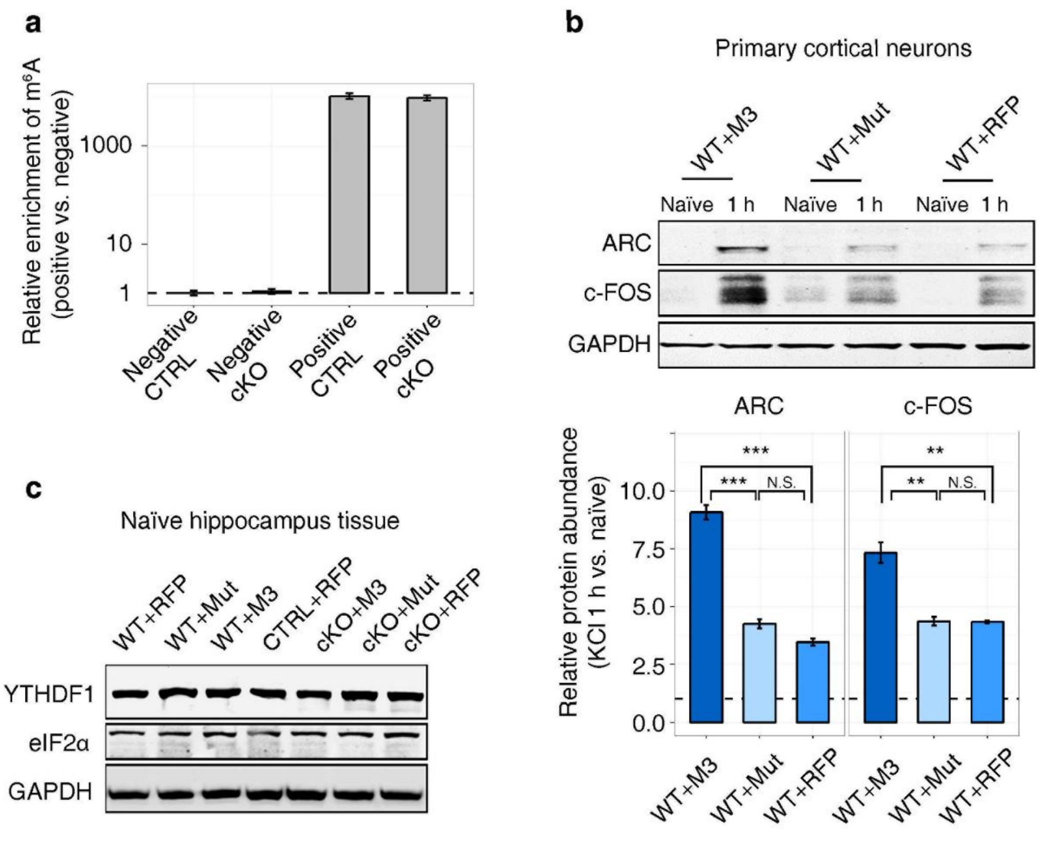

Fig. S7. Regulation of IEG protein translation by METTL3.

a Positive (synthesized m^{6}A RNA) and negative (control RNA, see Methods) controls of MeRIP-qPCR experiments (related to Fig. 5a). **b** Overexpressing *Mettl3* in primary cortical neurons enhances translation of *Arc* and *c-Fos* upon KCl treatment. **c** Western blot assay of translation-related proteins. Student's *t*-test, $^{**}P < 0.01$, $^{***}P < 0.001$. N.S., not significant, $n = 3$ replicates.
