# Supplementary material for: METTL3-mediated N6-methyladenosine mRNA modification enhances long-term memory consolidation
Source: Cell Res. 2018 Oct 8;28(11):1050–61. doi: 10.1038/s41422-018-0092-9 (PMC6218447; doi:10.1038/s41422-018-0092-9)
Supplement: Supplementary file 8 — Supplementary information, Figure S8 [file 41422_2018_92_MOESM8_ESM.pdf]

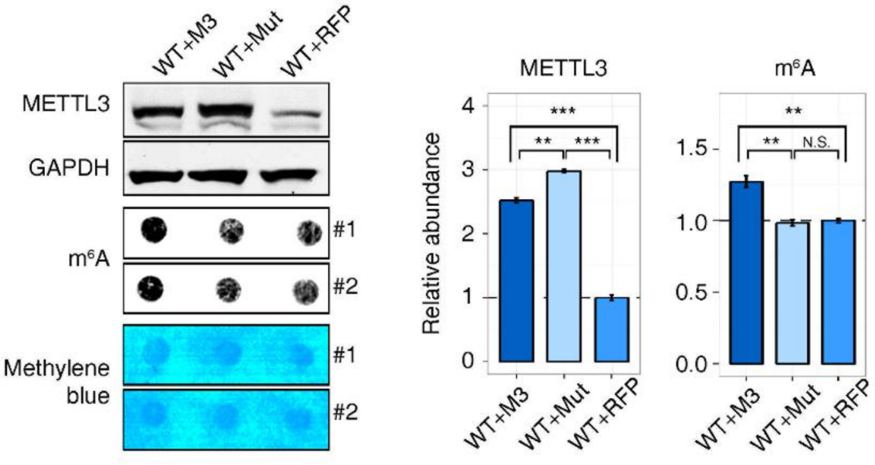

Fig. S8. Overexpression of *Mettl3* in hippocampus.

Overexpressing *Mettl3* in mouse hippocampus increases m^{6}A abundance. #1 and #2 represent two biological replicates.
